# Absolute Stereochemistry Determination of Bioactive Marine-Derived Cyclopeptides by Liquid Chromatography Methods: An Update Review (2018–2022)

**DOI:** 10.3390/molecules28020615

**Published:** 2023-01-07

**Authors:** Carla Fernandes, Ricardo Ribeiro, Madalena Pinto, Anake Kijjoa

**Affiliations:** 1 Laboratório de Química Orgânica e Farmacêutica, Departamento de Ciências Químicas, Faculdade de Farmácia, Universidade do Porto, Rua de Jorge Viterbo Ferreira 228, 4050-313 Porto, Portugal; 2 Interdisciplinary Centre of Marine and Environmental Research (CIIMAR), Edifício do Terminal de Cruzeiros do Porto de Leixões, Avenida General Norton de Matos s/n, 4050-208 Matosinhos, Portugal; 3 ICBAS-Instituto de Ciências Biomédicas Abel Salazar, Universidade do Porto, Rua de Jorge Viterbo Ferreira 228, 4050-313 Porto, Portugal

**Keywords:** absolute configurations, biological activity, chiral derivatizing agent, chiral stationary phase, liquid chromatography, Marfey’s method, marine resources, cyclopeptides, stereochemistry

## Abstract

Cyclopeptides are considered as one of the most important classes of compounds derived from marine sources, due to their structural diversity and a myriad of their biological and pharmacological activities. Since marine-derived cyclopeptides consist of different amino acids, many of which are non-proteinogenic, they possess various stereogenic centers. In this respect, the structure elucidation of new molecular scaffolds obtained from natural sources, including marine-derived cyclopeptides, can become a very challenging task. The determination of the absolute configurations of the amino acid residues is accomplished, in most cases, by performing acidic hydrolysis, followed by analyses by liquid chromatography (LC). In a continuation with the authors’ previous publication, and to analyze the current trends, the present review covers recently published works (from January 2018 to November 2022) regarding new cyclopeptides from marine organisms, with a special focus on their biological/pharmacological activities and the absolute stereochemical assignment of the amino acid residues. Ninety-one unreported marine-derived cyclopeptides were identified during this period, most of which displayed anticancer or antimicrobial activities. Marfey’s method, which involves LC, was found to be the most frequently used for this purpose.

## 1. Introduction

Since more than two-thirds of the global surface area are covered by oceans, marine organisms represent more than half of the total biodiversity [1]. Therefore, oceans constitute a rich source of many unique and novel compounds [2,3]. Long-term evolution of marine organisms promoted the fruitful development of a great number of natural products when exposed to extreme conditions, such as pressure, light, temperature, and salinity [4,5]. From 2015 to 2018, 17 clinically available drugs, based on marine natural products or their derivatives, were reported being 28 drugs in Phases I–III clinical trials [6]. Since 2018, great efforts have been made to find new therapeutically potent chemical and biological entities and, currently, 68 patents from marine organisms have been filed [7].

Among a large number of metabolites from marine-derived organisms, peptides are of pivotal relevance due to their unique structures, chemical properties, and interesting biological effects [8,9]. They are found in marine tunicates [10,11], sponges [12,13,14], algae [15,16], bacteria [17,18,19], cyanobacteria [20,21,22], fungi [23,24,25], and other invertebrates [26]. Several marine-derived peptides are obtained from symbionts [27], as well as non-symbiotic microorganisms such as sponge-associated fungi [28,29].

**Figure 1:**
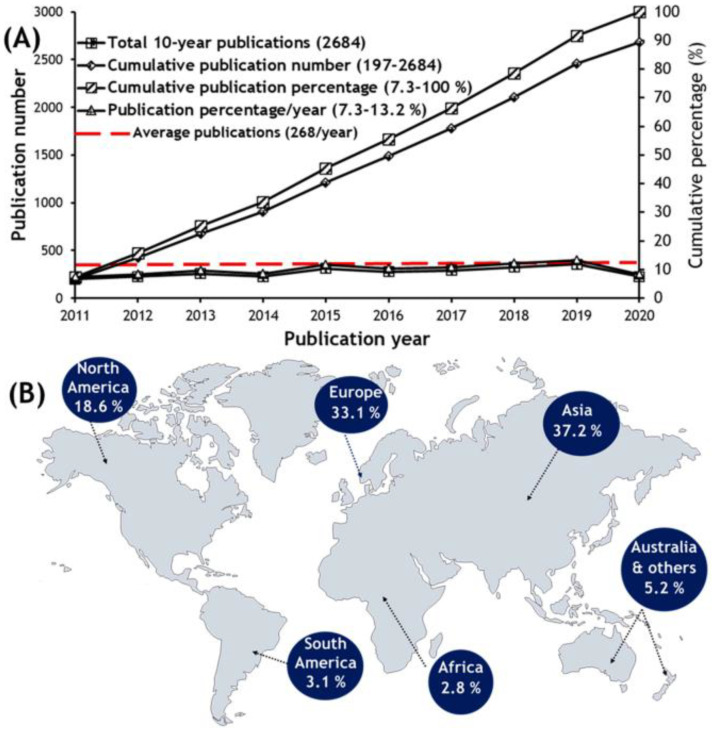
(**A**) Research trend on marine derived peptides from 2011 to 2020, and (**B**) the global distribution in terms of publication number and percentage. (Reprint with permission from [42], Copyright (2021) ELSEVIER).

Marine-derived peptides display a broad spectrum of bioactivities [30,31,32], such as antibacterial, antifungal, antiviral, antitumor, antioxidant, antihypertensive, antiatherosclerotic, anticoagulant, antidiabetic, analgesic, immunomodulatory, neuroprotective, and appetite-suppressing activities, which are attracting the increasing attention of pharmaceutical [33,34], cosmeceutical [35,36], and nutraceutical [37,38,39] industries. Two marine peptide-derived drugs have reached the market: ziconotide [40], a synthetic version of the *N*-type calcium channel blocker ϖ-conotoxin MVIIA for the treatment of patients suffering from chronic pain, and brentuximab vedotin (Adcetris^®^), a synthetic derivative of dolastatin 10 linked to an antibody for the treatment of Hodgkin’s lymphoma [41]. Moreover, marine-derived peptides constitute one of the research topics that provides a very high output, with a huge increase in the number of publications (268 per year) from 2010 to 2020 (Figure 1A), with both Asia (37.2%) and Europe (33.1%) as the major contributors (Figure 1B), and this trend is expected to continue [42].

The remarkable properties of cyclopeptides, such as low inherent toxicity, good affinity, and high selectivity for protein targets [43], make them attractive molecules to be explored. Their structures may comprise diverse structural scaffolds, usually non-proteinogenic amino acids [44], or with some modifications such as methylation [45], sulfuration [46], lipidation, and acetylation [47]. These characteristics play a crucial role in the interactions with biotargets [48,49]. Nevertheless, not all groups of marine organisms produce cyclopeptides with all these characteristics, and some are more common in certain groups. For example, cyclopeptides found in marine sponges such as *Lithistid* and *Theonella* are characterized by a high proportion of d- and/or *N*-methylated amino acids originating from symbiotic microorganisms [50,51,52,53,54]. Bacteria incorporate non-proteinogenic amino acids to prevent the proteolysis of peptides through stabilization of the backbone conformation and/or by elimination of the enzyme recognition site [55,56,57,58]. Secondary structures, especially the *α* helix, are common in antimicrobial marine peptides [59,60,61,62] because the presence of the *α* helix allows their penetration through a microbe’s membrane [60,61].

Cyclodepsipeptides—molecules where one or more amide groups are replaced by the corresponding ester—are often found in marine organisms [63,64,65]. Depsipeptides are biosynthesized by non-ribosomal peptide synthetases in combination with either polyketide synthase [66,67,68] or fatty acid synthase enzyme systems. This type of bioactive peptide has also contributed to revolutionizing the peptide chemical space [69]. Proline-rich cyclopeptides also play a crucial role in drug discovery that may provide useful scaffolds for modulating more challenging biological targets, such as protein–protein interactions and allosteric binding sites [70]. Lipodepsipeptides, which contain a lipid moiety linked to a peptide portion, are also well distributed among marine organisms and have shown different biological activities, such as phytotoxic, antimicrobial, immunosuppressive, cytotoxic, hemolytic, among others. Their bioactivities seem to be related to the interactions between a hydrophobic segment with the plasma membrane because they mimic the architecture of the membranes [71].

As one of the richest sources of structurally diverse bioactive compounds, considerable attention has been drawn towards the production and bioactivity of marine cyclopeptides [42]. Moreover, in recent decades, advanced synthetic methods have been applied to overcome supply problems of marine peptides of interest, affording higher quantities of compounds required for large-scale biological assays [72,73,74,75,76]. In addition to classical solid-phase and solution-phase techniques of peptide synthesis [77], significant efforts have been carried out for the introduction of sustainable and innovative processes for synthesis and purification methodologies [78,79]. Marine cyclopeptides are also interesting models for molecular modifications to obtain more potent compounds with improved properties [80,81]. For example, non-proteinogenic amino acids can be incorporated to prevent the proteolysis of peptides [81]. Acetylation of the *N*-terminus of short peptides has shown to improve peptidase stability in serum, and hence, increases their half-life [82]. Protein glycosylation can increase protein–protein interaction and protein stability. Interestingly, the glycosylation of peptides is suggested to improve peptide permeability [83], increase metabolic stability, and lower clearance rate, thereby improving bioavailability [84]. Peptide half-life is expanded through lipidation, where a long-chain saturated lipid is acylated to an amino acid to facilitate its binding to a carrier serum protein [85,86].

Cyclopeptides isolated from marine sources can comprise a different number of amino acid subunits, and the assignment of their absolute configurations is an absolute requirement for complete characterization [87]. Their physical, chemical, and biological/pharmacological properties are closely related to their absolute configurations, and stereochemical assignments continue to be a great challenge for natural products and medicinal chemists [88].

Currently, different tools, such as X-ray crystallography [89,90], vibrational circular dichroism [91,92,93], optical rotatory dispersion [94], electronic circular dichroism [95,96,97], nuclear magnetic resonance (NMR) [98,99,100], Marfey’s method in combination with liquid chromatography (LC) [101,102,103,104], and chiral LC [105,106,107], can be used to determine the absolute configurations. In some cases, such as lipopeptides, additional stereogenic centers are present in the lipid chain that might require additional experiments, such as Mosher’s method, or others [108,109].

A literature survey from 1996 to 2017 covering all the reports on chromatographic methods for the absolute stereochemistry determination of marine peptides revealed that Marfey´s method and/or chiral LC are very efficient and widely used [110]. Moreover, 52% of the reports described the use of Marfey’s method, 21% employed chiral LC analysis, and 27% included the application of both methods. In some cases, a combination of both methods are found to be necessary for the absolute configurations assessment of all the amino acid residues [110]. In addition, LC methods are also widely used for the assignment of the absolute configurations of peptides isolated from natural sources other than marine organisms [111,112,113,114].

In a continuation of our previous publication [110] and to analyze current trends, herein, recent studies concerning new bioactive marine cyclopeptides are analyzed regarding the absolute stereochemistry determination of the amino acid residues by LC methodologies. The chemical structures and biological/pharmacological activities of the marine-derived peptides are also described to provide an update for this field of research. Structural issues relevant to the biological/pharmacological activities are also emphasized when considered pertinent.

## 2. Liquid Chromatography Methods for Determination of Absolute Configurations of Peptides

LC is an indispensable technique in several research laboratories all over the world, especially when combined with different detectors such as ultraviolet-visible (UV-Vis), mass spectrometry (MS), and fluorescence detection [115]. LC is a method of choice associated to both Marfey´s and chiral LC methods [110].

**Figure 2:**
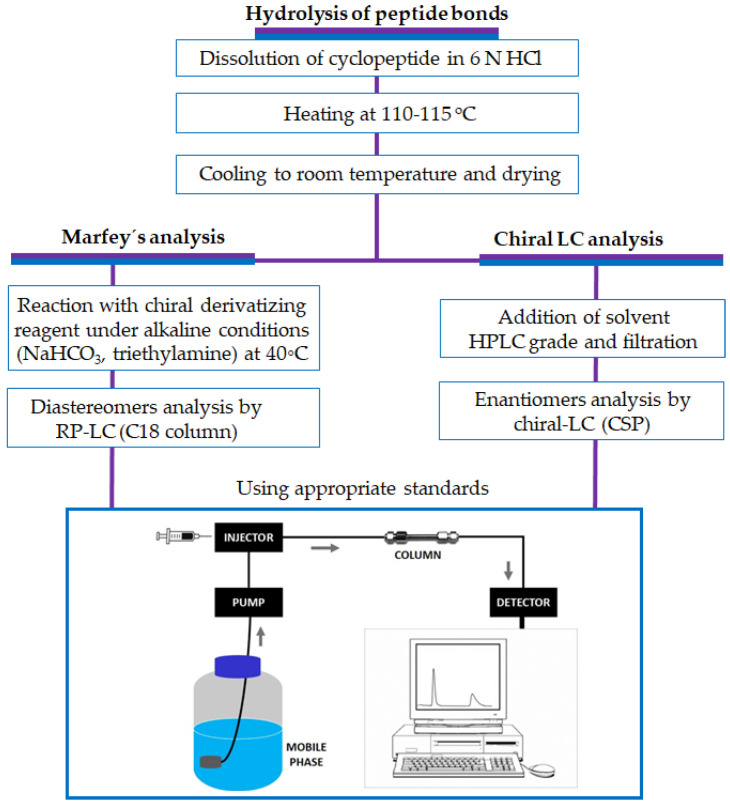
The steps generally used to determine the configurations of the amino acid residues of peptides by LC methods. RP-LC: reversed-phase liquid chromatography; HPLC: high-performance liquid chromatography; CSP: chiral stationary phase.

The first step, in both methods, to determine the absolute configurations of the amino acids of marine peptides is a total or partial hydrolysis of peptide bonds to obtain individual amino acid residues (Figure 2). Typically, the hydrolysis reaction is carried out in acidic condition using 6 N of HCl solution at 110–115 °C for 24 h [110].

**Figure 3:**
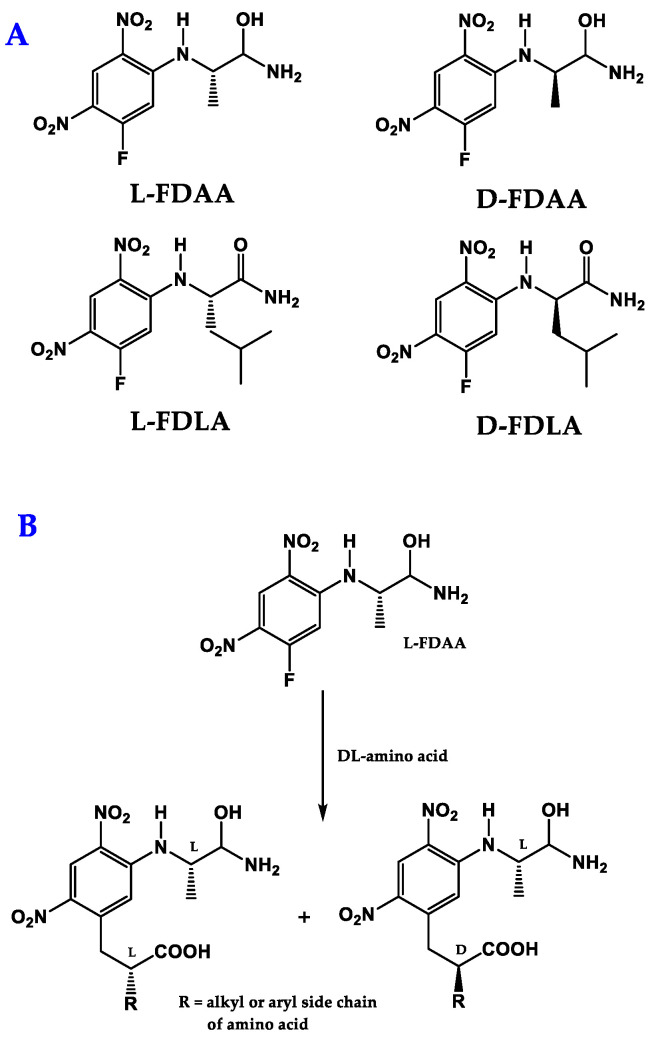
Chemical structures of both enantiomers of 1-fluoro-2,4-dinitrophenyl-5-d,l-alanine amide (FDAA) and 1-fluoro-2,4-dinitrophenyl-5-d,l-leucine amide (FDLA) derivatizing agents (**A**); reaction of d/l-amino acids (dl-aa) with l-FDAA to give a pair of diastereomers (**B**).

In Marfey´s method, after the acidic hydrolysis of peptides, the amino acid residues are derivatized with suitable chiral derivatizing reagents (CDRs). The reagents 1-fluoro-2,4-dinitrophenyl-5-d,l-alanine amide (FDAA) and 1-fluoro-2,4-dinitrophenyl-5-d,l-leucine amide (FDLA) (Figure 3A) are the most widely used [110].

The CDRs react stoichiometrically, without racemization, under gentle heating at 40 °C within 1–2 h in alkaline condition, with the amino group of l- and d-amino acids residues yielding diastereomers (Figure 3B) [116]. It is important to mention that this method was found to be ineffective for some amino acid residues, especially those containing ring- and α-methyl-substituted phenylalanine and phenylalanine amides [117]. Nevertheless, a new approach, called the “*O*-Marfey method”, was described for the derivatization of α-hydroxy acids with l-FDAA by increasing the nucleophilicity of the hydroxyl group for the reaction to take place [118]. This method requires the addition of NaH (60% dispersed in oil) to a solution of α-hydroxy acids in tetrahydrofuran at room temperature. Then, the CDR (l-FDAA) is added to this solution and stirred under argon or nitrogen atmosphere [118]. In other cases, there is a need for optimization, such as by using a solution of FDAA in acetone, as well as a higher reagent concentration and longer reaction times [119]. Of note is that diastereomers can also be obtained by microwave-assisted synthesis [120].

The synthesis of FDAA as a CDR to determine the stereochemistry of amino acids was described for the first time by Peter Marfey in 1984 [121]. FDAA was obtained from difluoro dinitro benzene by substitution of one of its two fluorine atoms by l-alanine amide. Then, a nucleophilic substitution of the remaining reactive aromatic fluorine was performed with the free amino group on l- and d- alanine. From this reaction, diastereomeric pairs were obtained, which were separated by RP-LC. Amide was chosen because it is quite stable and neutral and does not undergo racemization [121]. Now, FDAA is universally known as Marfey’s reagent and continues to be widely used. Over the years, other structural variants of FDAA have been developed [122].

Generally, after the derivatization reaction, the diastereomers are analyzed by an RP-LC, using suitable standards for comparison to accurately determine the absolute configurations of the peptides [119]. Additionally, other methodologies have also emerged, including LC-mass spectrometry (LC-MS), thin-layer chromatography (TLC), and gas chromatography (GC) [123,124]. The key advantages of Marfey´s method include improved detection sensitivity compared to underivatized amino acids, the commercial availability of both enantiomers of FDAA, and the use of readily available LC technology.

Harada et al. [125] proposed a method named “advanced Marfey’s method”. This method uses LC-MS to determine the absolute configurations of the amino acid residues. Since this method does not necessarily require amino acid standards to detect and identify target amino acids, it can be used to determine the absolute configurations of unusual amino acids for which standards are not available. In this method, the absolute configuration of an amino acid is deduced from its LC retention times of the l-FDLA derivative of the original amino acid, and its enantiomer formed by racemization. Therefore, the racemization is essential for this method [125]. Later, the same group introduced “dl-FDLA derivatization”, a procedure that does not require the conventional chemical racemization process [126]. Due to this d,l-FDLA system, the advanced Marfey’s method has been improved to be simple, more rapid and reliable, and its applicability has been significantly extended [103,104].

In order to develop a highly sensitive analytical Marfey’s method capable of rapidly and unambiguously determining the absolute configurations and enantiomer regiochemistry for all commonly found amino acids, Vijayasarathy et al. [102] developed the C3 Marfey’s method. This method is a more refined variation on the existing Marfey’s method, using the same CDRs (l-FDAA and d-FDAA) but with a C3 LC column held at 50 °C, and a H_2_O/MeOH gradient elution modified by isocratic 1% HCO_2_H in CH_3_CN, and taking advantage of both UV (340 nm) and (±)-ESIMS detection. This method was shown to be very effective, allowing the stereochemical assignments of all amino acids, including isoleucine stereoisomers. Moreover, the residual Marfey’s reagent did not obscure any target amino acid [102].

The applicability and limitations of Marfey’s method and its optimized methods have been extensively examined in several reviews [116,119,122]. These methods were also found to be promising for applications in several other research fields and for other chiral molecules/drugs [122].

Regarding the chiral LC method, determination of the absolute configurations is developed by a direct analysis of the amino acid residues present in the acid hydrolysate without prior derivatization. First, enantioseparation of standards amino acids is performed on a chiral stationary phase (CSP). Then, comparison and/or co-injection of hydrolysate with the amino acids standards is performed, under the same chromatographic conditions, leading to the identification of the amino acid constituents [110].

**Figure 4:**
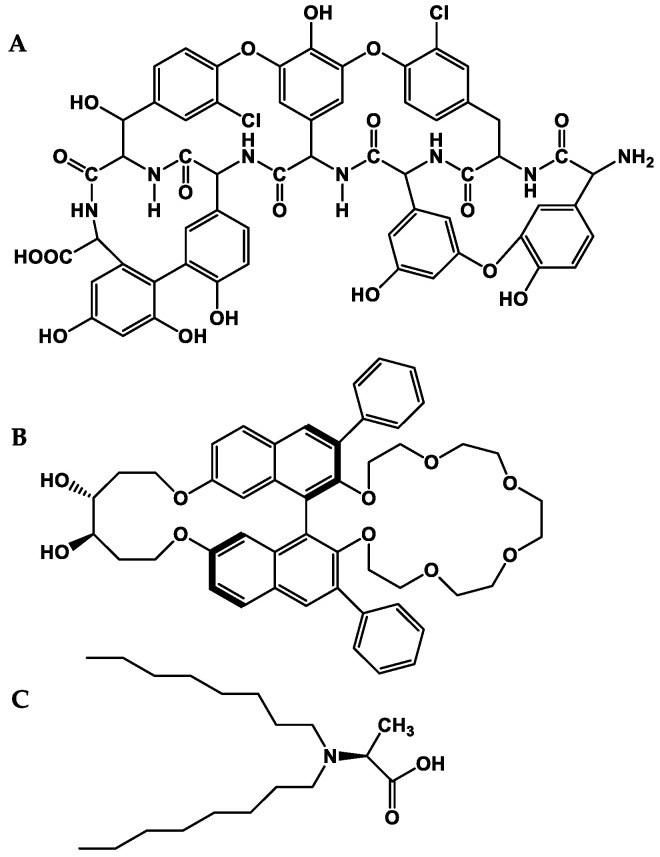
Chemical structures of macrocyclic antibiotic-based (**A**), crown ether-based (**B**), ligand exchange-type (**C**) chiral stationary phases.

The success of an efficient enantioseparation is mainly determined by the chiral discriminative power of the CSP. Over the years, various types of CSPs have been described [127,128,129]; nevertheless, the development of new CSPs for LC continued to be an evolutionary subject, encompassing the preparation of new chiral selectors [130], the introduction of new chromatographic supports [131], and the application of different synthetic approaches for the preparation of CSPs [132]. Macrocyclic antibiotic-based, crown ether-based, and ligand exchange-type (Figure 4) are the most-used CSPs for the enantiomeric separation of amino acids and primary amine-containing compounds [133,134,135,136]. Evidently, the same trend was observed for the stereochemical determination of the amino acids subunits of marine peptides [110].

A direct analysis of amino acid hydrolysates without further derivatization is the main advantage of chiral LC, since it is a faster and simpler procedure when compared to indirect methods. Its principal drawback is the high cost of the commercial chiral columns, which could be a hurdle for these analyses [137].

**Figure 5:**
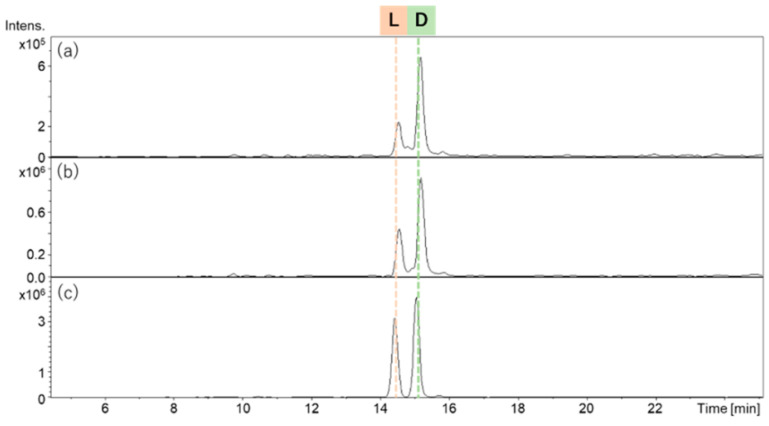
Time course of the racemization of lysine. Acid hydrolysate LDAA lysine derivatives: (**a**) 4 h hydrolysis; (**b**) 8 h hydrolysis; (**c**) 16 h hydrolysis. (Reprint with permission from [138], Copyright (2021) American Chemical Society).

Although these methods are highly effective, one limitation of both is that racemization can occur for some amino acid residues during the acid hydrolysis [138,139]. For example, the analysis of the acidic hydrolysate of a cyclopeptide isolated from a marine sponge, *Homophymia* sp., showed that the racemization of lysine took place. Although this peptide contains only one lysine residue, both d- and l-enantiomers were detected in almost equal amounts. By performing an analysis of the hydrolysates with shorter reaction times of 4 and 8 h, it was found that racemization occurred during acidic hydrolysis (Figure 5). The longer the reaction time, the higher the content of l-lysine, indicating a formation of this enantiomer by epimerization [138].

Other difficulties, such as the absence of commercially available standard amino acids, demanding time and synthetic efforts to prepare them [140], the existence of identical amino acids in the peptide skeleton [141], or amino acids with more than one chiral stereocenters [142], make elucidation a more difficult task. One strategy to solve this kind of problem is the use of synthesis, which in some cases, resulted in a revision of the proposed structures [139,143,144].

## 3. New Marine-Derived Cyclopeptides Reported from January 2018 to November 2022

There are several reports describing the isolation and biological activity evaluation of new marine-derived peptides during the period of January 2018 to November 2022. Among them, chromatographic methods—specifically Marfey’s method and chiral LC analysis—were used to assess the absolute configurations of 91 new marine-derived cyclopeptides (**1**–**91**). Table 1 summarizes the names and types of cyclopeptides, compound producers, chromatographic methods, the biological activities of each compound and their references. Several marine sources were described, including marine-derived bacteria, cyanobacteria, marine sponges, and marine-derived fungi, among others. The potential of marine-derived cyclic peptides **1**–**91** as anticancer and antimicrobial agents is expected based on previous reviews [145,146,147], which reinforces the marine organisms as a rich source of bioactive compounds for these therapeutic classes.

In this section, the marine-derived cyclopeptides are organized according to the main biological activity.

### 3.1. Anticancer Activity

**Figure 6:**
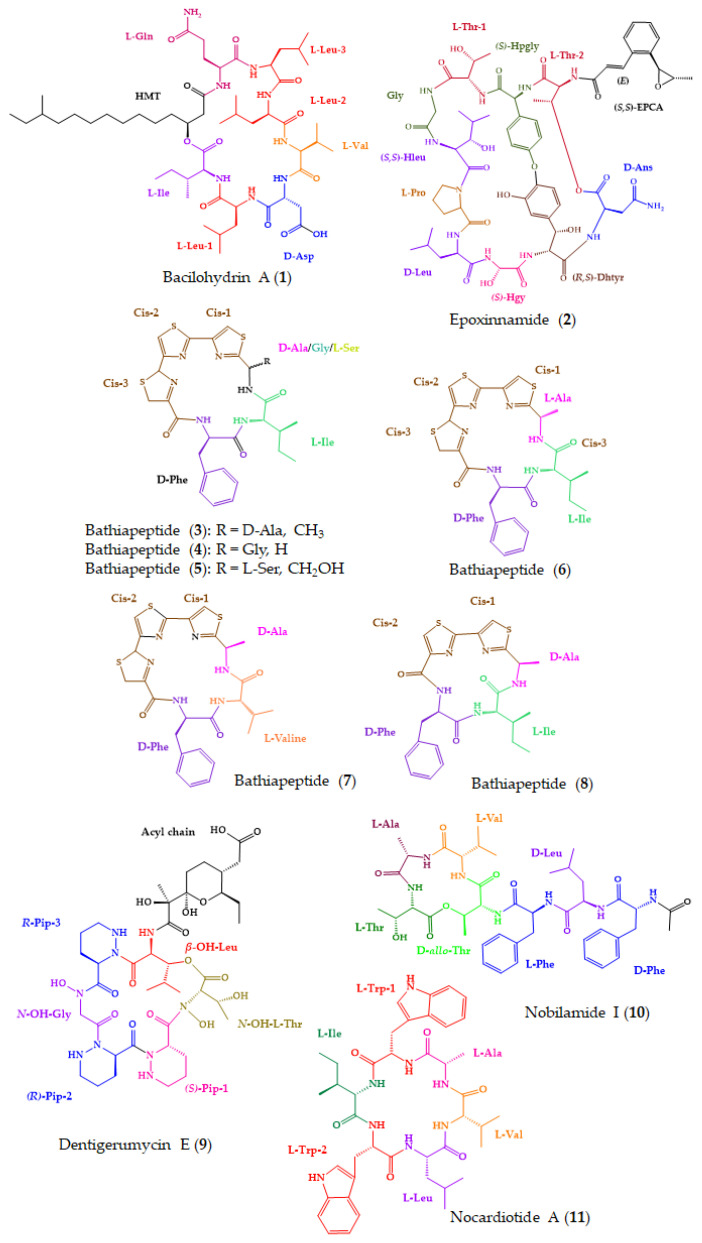
Chemical structures of marine-derived cyclopeptides with anticancer activity isolated from marine bacteria (**1**–**11**).

One of the most described biological activities associated to marine-derived cyclopeptides is anticancer activity [185]. Consistent with similar findings in previous works, several isolated marine peptides exhibited cytotoxicity. Among the 91 new marine-derived cyclopeptides, 43 showed promising results regarding anticancer activity and 11 (**1**–**11**) were isolated from marine-derived bacteria (Figure 6). Marine-derived bacteria have been shown to be a rich source of natural products, including cyclopeptides [71,186]. Bacterial natural products constitute 29% of Food and Drug Administration (FDA)-approved natural product-derived drugs, which mostly function as antitumor or antibacterial agents [187].

A new cyclic lipopeptide, bacilohydrin A (**1**), was isolated from a crude culture extract of *Bacillus* sp. SY27F, obtained from the Indian Ocean hydrothermal vent [148]. Compound **1** exhibited significant cytotoxicity against three cancer cell lines—specifically, DU-145, MCF-7, and HepG2—with IC_50_ values of 50.3–175.1 nM. The positive control, paclitaxel, showed IC_50_ values of 10.9–94.3 nM. The absolute configurations of the amino acids in **1** were determined by a modified Marfey’s method revealing the presence of seven mixed d/l-amino acids. It is important to highlight that it was the first report of a determination of the absolute configuration of a d-aspartic acid residue in the surfactin class [148].

The advanced Marfey’s method, in combination with ^3^*J*_HH_ and rotating-frame Overhauser effect spectroscopy (ROESY) analysis, DP4 calculation, and genomic analysis, was applied to establish the absolute configuration of the amino acid units of epoxinnamide (**2**), a new cinnamoyl-containing nonribosomal bicyclic decadepsipeptide (Figure 6), which was isolated from the culture extract of an intertidal mudflat-derived *Streptomyces* sp. OID44 [149]. Cinnamoyl-containing nonribosomal peptides represent a unique family of actinobacterial metabolites associated with diverse bioactivities [188]. Compound **2** induced quinone reductase activity in murine Hepa-1c1c7 cells, at 5 µM, by 1.6-fold. Additionally, **2** displayed considerable antiangiogenesis activity in human umbilical vein endothelial cells, with an IC_50_ of 13.4 µM. The positive control, sunitinib, showed an IC_50_ value of 1.7 µM [149].

A new family of polypeptides, bathiapeptides A1 (**3**), A2 (**4**), and B-E (**5**–**8**), isolated from a marine biofilm-derived *Bacillus* sp. B19-2, exhibited high cytotoxicity against MGC-803, Hep G2, MCF-7, and HeLa cell lines. Compound **3** exhibited the highest cytotoxicity in all cell lines, with IC_50_ values ranging from 0.5 to 4.1 µM. The advanced Marfey’s method was able to establish the absolute configuration of isoleucine and phenylalanine of **3**–**8** [150]. Due to the presence of a rare polythiazole moiety in **3**–**8** (Figure 6), the thiazole-derived alanine moiety could not be directly determined by Marfey’s method (or chiral LC) since the epimerization of thiazole-based amino acids can occur during acid hydrolysis [189]. To solve this issue, ozonolysis was carried out to break the thiazole ring before derivatization by Marfey’s reagents [190]. Moreover, since Marfey’s method does not allow distinguishing l-isoleucine from l-allo-isoleucine [104], an X-ray analysis was performed to prove the structure of l-isoleucine [150].

Dentigerumycin E (**9**), a new cyclic hexapeptide containing three piperazic acids and a pyran-bearing polyketide acyl moiety (Figure 6), was isolated from the extract of a coculture of marine-derived *Streptomyces* and *Bacillus* strains, which were simultaneously isolated from an intertidal mudflat. The absolute configurations of the amino acid residues of **9** were established by a combination of advanced Marfey’s method with ROESY correlations. Compound **9** exhibited moderate antiproliferative and antimetastatic activities against several human cancer cell lines, including A549, HCT-116, MDA-MB-231, SK-HEP-1, and SNU638, with IC_50_ values ranging from 27 to 39 μM. The positive control, etoposide, showed IC_50_ values ranging from 0.42 to 6.21 µM. Compound **9** was also assayed against MCF-10A to determine its cancer cell-specific cytotoxicity and showed an IC_50_ value higher than 50 µM, indicating that **9** did not exhibit significant cytotoxicity against normal human epithelial cells. Additionally, **9** also showed antimetastatic potential against MDA-MB-231 in wound healing and cell invasion assays. In the wound healing assay, **9**, at 20 and 40 µM, inhibited cell migration by 20 and 48%, respectively, while in the cell invasion assay, **9** exhibited inhibitory activity by 10 and 34% at 20 and 40 µM, respectively. A structure–activity relationship (SAR) study suggested that 2-*N*-OH, 16-*N*-OH, and 37-OH (carboxylic acid) in **9** are essential for its antiproliferative and antimetastatic activities [151].

Another promising compound with anticancer activity is a cyclic depsipeptide, nobilamide I (**10**), isolated from the saline cultivation of a marine-derived bacterium, *Saccharomonospora* sp. strain CNQ-490, obtained from a 45 m deep-sea sediment sample at 2 km west of the Scripps pier, La Jolla Canyon, in California. Nobilamide I (**10**) decreased cancer cell motility by inhibiting epithelial-mesenchymal transition (EMT) effectors. At a concentration of 5 μM, **10** inhibited migrations of AGS, A549, and Caco2 cells by ca. 70, 50, and 60%, respectively, and their invasions by ca. 60, 30, and 60%, respectively. It was suggested that **10** modulated the protein and mRNA expression levels of EMT N-cadherin and E-cadherin markers by downregulating the transcription factors, Snail, Slug, and Twist. In addition, **10** modulated the protein and mRNA expression levels of the matrix metalloproteinase family (MMP2 and MMP9) in the three cell lines. The absolute configurations of the amino acid residues were assigned by using the C3 Marfey’s analysis [152].

A new cyclic hexapeptide, nocardiotide A (**11**) (Figure 6), was isolated from a broth culture of an actinomycete, *Nocardiopsis* sp. UR67, associated with the marine sponge, *Callyspongia* sp., which was collected from the Red Sea, Ras Mohamed, Sinai, Egypt. The planar structure of the compound was elucidated by HRESIMS and a 1D and 2D NMR spectral analysis. The absolute configurations of the amino acid residues were solved by Marfey’s method. Compound **11** displayed significant cytotoxicity towards human MM.1S multiple myeloma, human HeLa cervix carcinoma, and murine CT26 colon carcinoma cell lines, with IC_50_ values of 8, 11, and 12 µM, respectively [153].

**Figure 7:**
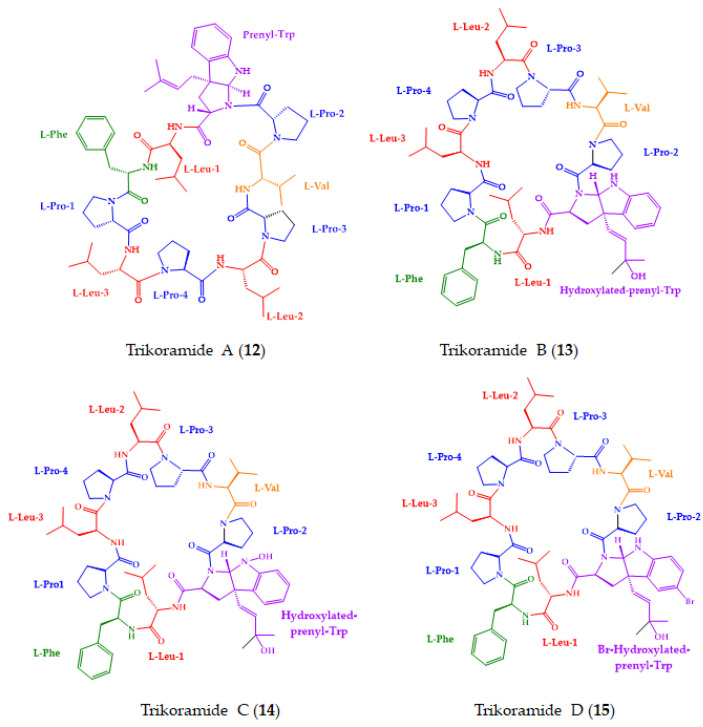

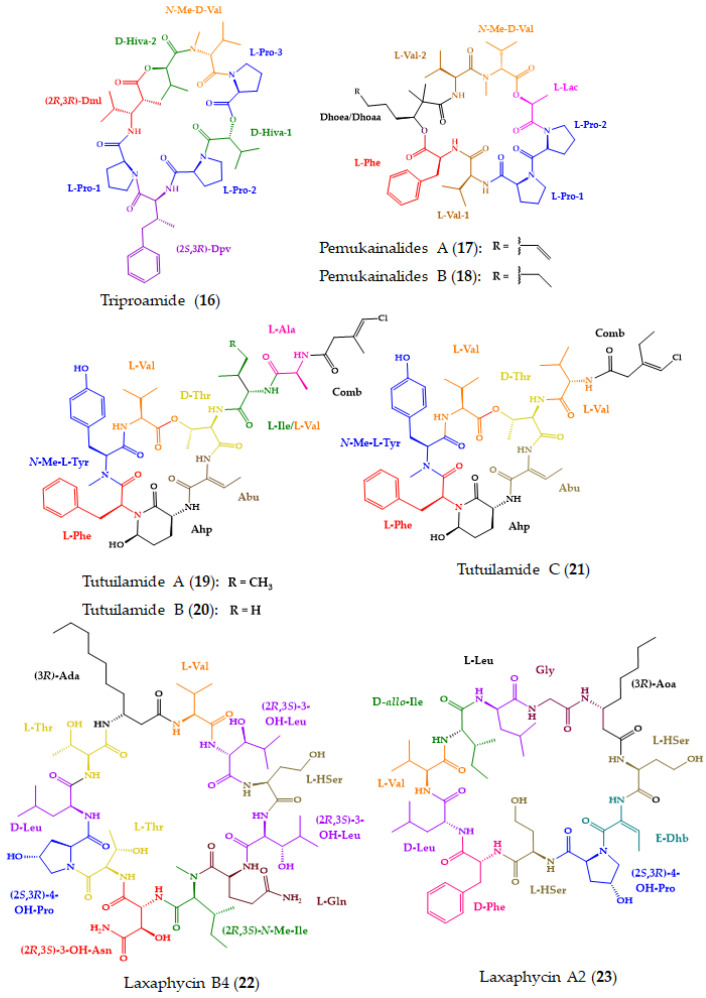
Chemical structures of marine-derived cyclopeptides with anticancer activity isolated from marine cyanobacteria (**12**–**23**).

Cyanobacteria are also an important source of new marine-derived cyclopeptides [191,192]. Compounds **12**–**23** (Figure 7) with anticancer activity were discovered in 2018.

Phyo et al. [154] described the isolation of a new cyclic decapeptide, trikoramide A (**12**) (Figure 7), isolated from samples of the marine cyanobacterium, *Symploca hydnoides*, which was collected from the intertidal shores of Trikora beach, Bintan Island, Indonesia. Compound **12** is a C-prenylated cyclotryptophan-containing cyanobactin whose absolute configuration was determined by Marfey’s method and a NOESY correlations analysis. Compound **12** displayed cytotoxicity against the MOLT-4 and AML2 cancer cell lines, with IC_50_ values of 4.8 and 8.2 μM, respectively [154].

Later, the same group isolated another three cyanobactins, trikoramides B–D (**13**–**15**) (Figure 7), from the same samples. Compounds **13**–**15** are analogues of **12**. The main difference in their structures is in the C-prenylated cyclotryptophan unit. Marfey’s method, in combination with the ECD spectra and NOESY correlations, were applied to determine their absolute structures. Compounds **13** and **15** showed cytotoxicity against the MOLT-4 cell line, with IC_50_ values of 5.2 µM and 4.7 µM, respectively [155].

Very recently, the same group further isolated three new cyclic depsipeptides, triproamide (**16**), and pemukainalides A (**17**) and B (**18**) (Figure 7), from the same cyanobacterium, which was collected from the same location. Compound **16** features a unique structure containing a rare 4-phenylvaline and a β-amino acid, dolamethylleucine. Stereochemical analyses were carried out by utilizing a combination of Marfey’s method, *J*-based configuration, and chiral LC analyses. For the absolute configuration of the 2-hydroxyisovaleric acid unit in **16**, a chiral column was used with a ligand exchange-type CSP to establish the *R*-configuration. Compound **17** exhibited cytotoxicity against the MOLT-4 leukemia cell line, with an IC_50_ value of 5.6 μM. The positive control, dolastatin, showed an IC_50_ value of 2.5 µM [156].

By using mass spectrometry-guided fractionation, together with molecular networking, Keller et al. [157] described the isolation of tutuilamides A (**19**) and B (**20**) (Figure 7), from the marine cyanobacterium, *Schizothrix* sp., which was collected from the main island of Tutuila in American Samoa, and tutuilamides C (**21**) (Figure 7), from a cyanobacterium, *Coleofasciculus* sp., which was collected from Palmyra Atoll. The structures of **19**–**21** contain a novel vinyl chloride-containing residue and several unusual residues, including 3-amino-6-hydroxy-2-piperidone and 2-amino-2-butenoic acid. Marfey’s method was used to determine the absolute configurations of their amino acid residues. Compounds **19**–**21** exhibited a moderate potency of cytotoxicity against H-460 lung cancer cell line. Interestingly, **19**–**21** displayed potent elastase inhibitory activity. Additionally, the binding mode to elastase was analyzed by X-ray crystallography, which revealed a reversible binding mode. Moreover, the amino acid backbone of the flexible side chain of **19** can establish an additional hydrogen bond in the elastase binding pocket, allowing enhanced inhibitory activity [157].

Two new cyclic lipopeptides, laxaphycins B4 (**22**) and A2 (**23**) (Figure 7), were isolated, along with the known peptide laxaphycin A, from the marine cyanobacterium, *Hormothamnion enteromorphoides*, which was collected at Garden Key, Dry Tortugas National Park [158]. Compound **22** displayed growth inhibition against HCT-116 cell line with an IC_50_ of 1.7 µM, and a synergistic effect to inhibit the growth of this cancer cell line when **22** was used in combination with laxaphycin A. The absolute stereochemistry of the amino acid residues of **22** and **23** was determined by both chiral LC using a macrocyclic antibiotic-based CSP, and by advanced Marfey’s method. In addition to those methods, 1D and 2D ROESY correlations were used to determine the geometric configuration of α,β-dehydro-aminobutyric acid [158].

**Figure 8:**
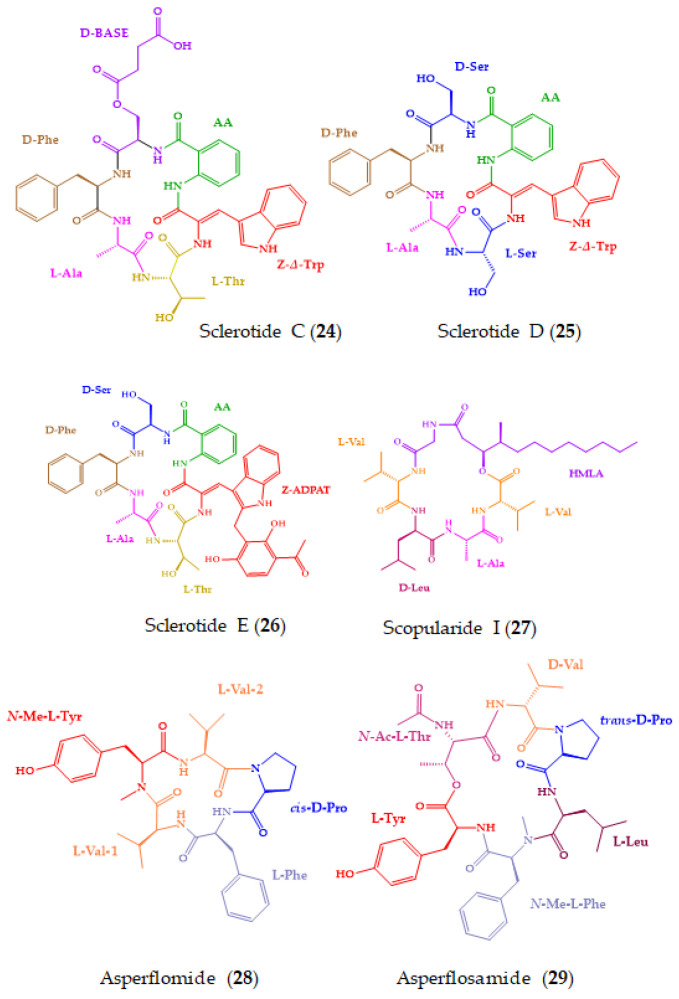
Chemical structures of marine-derived cyclopeptides with anticancer activity isolated from marine-derived fungi (**24**–**29**).

Cyclopeptides have also been isolated from a myriad of marine-derived fungi, mainly from genera *Aspergillus*, *Acremonium*, *Fusarium*, and *Penicillium* [193]. Marine-derived fungi represent a potential source for bioactive natural products, and an increasing number of new fungal metabolites, including peptides, have been discovered over the past years [194,195]. In addition, peptides isolated from the association of fungi with other macroorganisms produced a valuable and extra-large chemical database, exhibiting relevant biological activities [196]. From 2018, six marine-derived cyclopeptides (**24**–**29**) with anticancer activity were isolated from marine-derived fungi (Figure 8).

Sclerotides C–E (**24**–**26**) (Figure 8), new cyclohexapeptides, and scopularide I (**27**), a new lipodepsipeptide, were discovered from the fermented rice cultures of *Aspergillus sclerotiorum* SCSIO 41031, isolated from a soft coral, which was collected in Beihai, Guangxi Province, China. Among the isolated compounds, only **27** showed cytotoxicity towards human nasopharyngealcarcinoma cell lines, HONE1 and HONE1-EBV, with IC_50_ values of 13.0 and 10.1 µM, respectively (sorafenib was used as a positive control). Compound **27** also displayed moderate inhibition of acetylcholinesterase, with an IC_50_ value of 15.6 µM (tacrine was used as a positive control). The absolute configurations of the amino acid residues in **25** and **26** were established by Marfey’s method, while those of **24** and **27** were determined by a single crystal X-ray diffraction analysis [159].

Two new *N*-methylated cyclopeptides, asperflomide (**28**) and asperflosamide (**29**) (Figure 8), were obtained from the culture extract of *Aspergillus flocculosus* 16D-1, isolated from the inner tissue of the marine sponge, *Phakellia fusca*, which was collected from Yongxing Island, China. The planar structures of **28** and **29** were elucidated by the interpretation of HRESIMS and extensive analysis of 1D and 2D NMR spectra. The absolute configurations of the amino acid constituents in **28** and **29** were established by Marfey’s method, using UPLC-HRMS for analysis. Although **28** and **29** did not exhibit cytotoxicity against A2780, HCT-8, PC-9, SW480, MDA-MB-231, and U251 cancer cell lines, and nonmalignant cells (human cardiomyoblast cell lines HaCaT and CCD-18Co), they showed weak tankyrase1/2 inhibitory activity at the centration of 40 µM (XAV939 was used as a positive control) [160]. Tankyrase is a member of the poly(ADP-ribose)polymerase family which mediates Wnt signal transduction and has emerged as a new molecular target for the therapy of different kinds of cancer. Consequently, tankyrase inhibitors are considered as promising therapeutics for cancer treatment [197].

**Figure 9:**
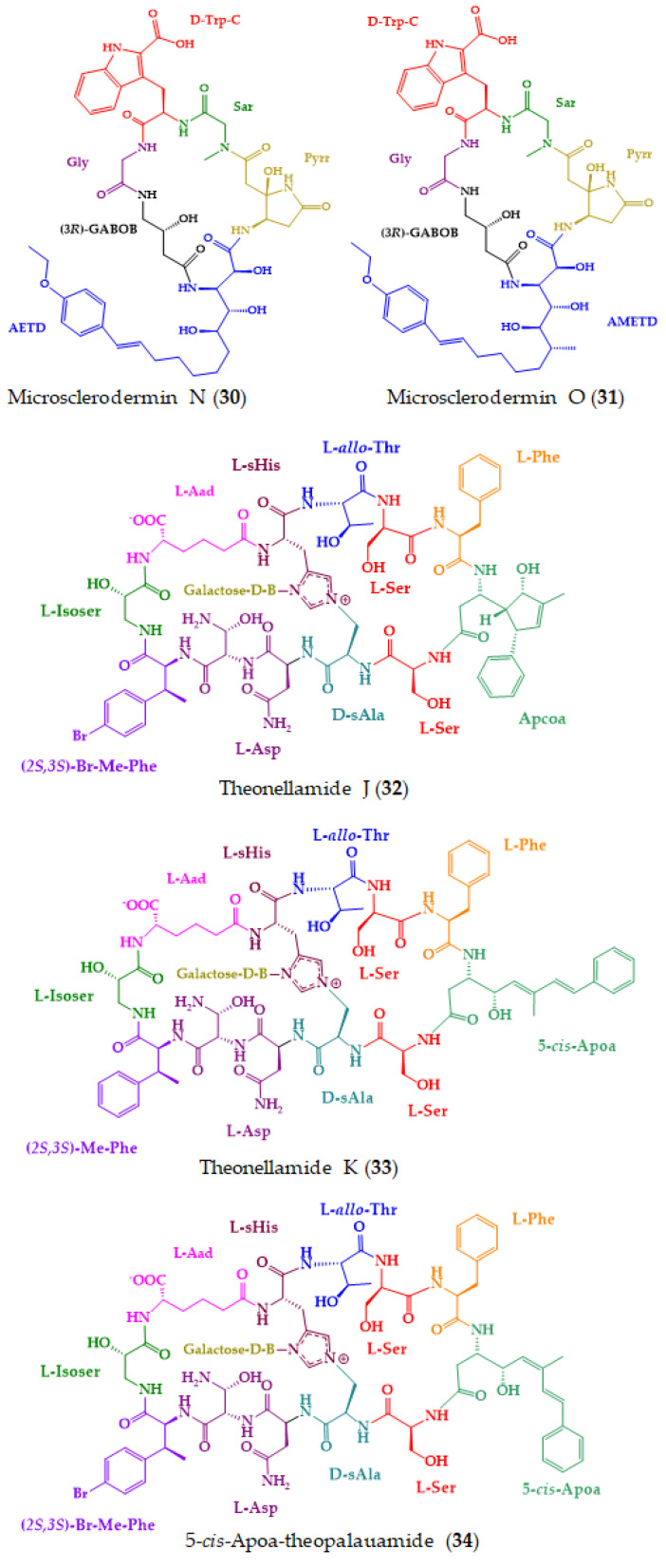

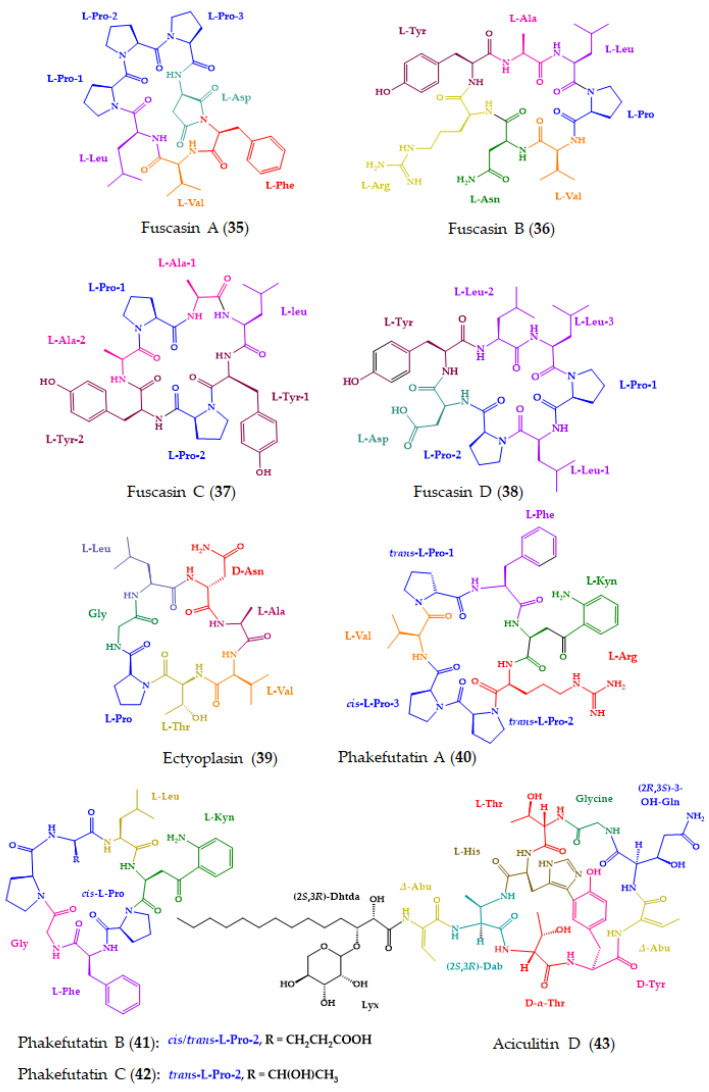
Chemical structures of marine-derived cyclopeptides with anticancer activity isolated from marine sponges and marine sponge-associated microorganisms (**30**–**43**).

A number of peptides and depsipeptides with intriguing structures and interesting biological activities have been discovered from marine sponges [198,199], some of which were shown to be produced by symbiotic microorganisms [27]. From 2018, 14 marine-derived cyclopeptides (**30**–**43**) (Figure 9) were isolated from marine sponges and from sponge-associated microorganisms.

From a deep-sea marine sponge, *Pachastrella* sp., two new peptides, microsclerodermins N and O (**30** and **31**) (Figure 9), were isolated [161]. Compounds **30** and **31** have unique structures since they possess a *p*-ethoxyphenyl moiety instead of a typical *p*-methoxyphenyl moiety at the terminus of the ω-phenyl-polyhydroxylated β-amino acid unit. The absolute configurations of all the stereogenic centers were determined by Marfey’s method and were found to be identical with other microsclerodermins. For a stereochemical study, a naphthalene-bonded stationary phase was used. Relevant cytotoxic activity was observed for both compounds against HeLa cells, with an IC_50_ of 0.77 µM and 0.81 µM, respectively. The positive control, doxorubicin hydrochloride, showed an IC_50_ value of 0.27 µM [161].

Theonellamide J (**32**), 5-*cis*-Apoa-theopalauamide (**33**), and theonellamide K (**34**) (Figure 9) were isolated from a red sea sponge, *Theonella swinhoei*, which was collected in the southern part of the Gulf of Aqaba. Compounds **33** and **34** exhibited a growth inhibitory activity against HCT-116 cell line, with an IC_50_ of 21.8 and 3.5 µM, respectively. The positive control, cytochalasin D, showed an IC_50_ value of 0.8 µM. This finding suggested that the 3-amino-4-hydroxy-6-methyl-8-phenyl-5*E*,7*E*-octadienoic acid (Apoa) subunit is relevant for the activity. The absolute configurations of the amino acids were established by Marfey’s and advanced Marfey’s methods. The samples were first analyzed by Marfey’s method, which allowed the configurations of four amino acids to be elucidated, followed by LC-MS (advanced Marfey’s method) for the assignment of the stereochemistry of the remaining amino acids [162].

**Figure 10:**
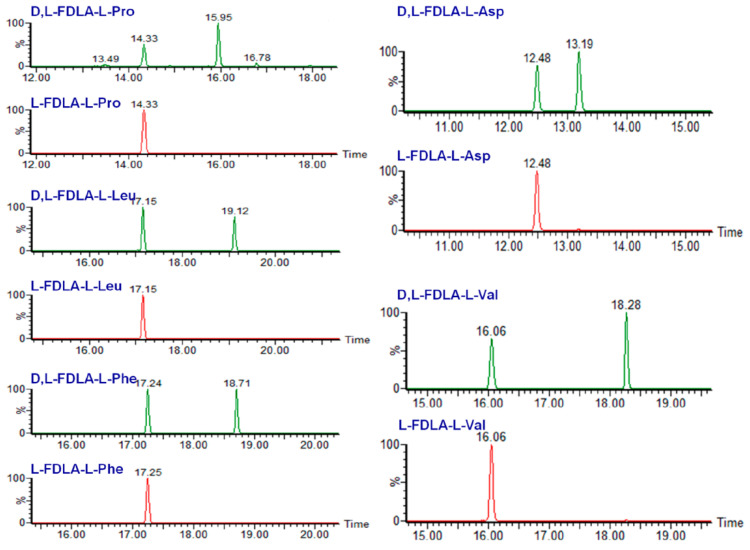
Advanced Marfey’s method coupled with a UPLC-HRMS analysis of fuscasin A (**35**), using the amino acid standards l-Pro, l-Ala, l-Val, l-Phe, l-Leu, l-Tyr, l-Arg, and l-Asp, which were treated with l-FDLA and d-FDLA. (Reprint with permission from [163], Copyright (2019) American Chemical Society).

Fuscasins A–D (**35**–**38**) (Figure 8) are new cyclic heptapeptides isolated from the marine sponge, *Phakellia fusca*, which was collected from the South China Sea. Compound **35** exhibited a remarkable cytotoxicity against HepG2 cells, with an IC_50_ value of 4.6 μM, while **36**–**38** were inactive even at a concentration up to 20 μM. Cisplatin was used as the positive control with an IC_50_ value of 4.2 μM. Interestingly, **35** did not exhibit cytotoxicity against the nonmalignant rat cardiomyoblast H9C2 cell line even at concentrations up to 100 μM, suggesting that this compound may have a selective growth inhibitory effect against HepG2 cells. Moreover, none of the compounds displayed cytotoxicity against NCI-H460, MCF-7, HeLa, PC9, and SW480 cell lines. Compound **35** is a structurally unique cyclic heptapeptide with a pyrrolidine-2,5-dione-bearing backbone. The absolute configurations of amino acid residues were determined by the advanced Marfey’s method by derivatizing its acid hydrolysis products with l-FDLA, followed by analysis with UPLCESI-QTOF MS. Figure 10 shows the chromatograms obtained after appropriate selective ion monitoring channels of the amino acid standards (green peaks) and Marfey’s derivatives (red peaks) for comparison of the retention times [163].

Marfey’s method was also used to determine the absolute configurations of the amino acid residues of a new cyclic heptapeptide, ectyoplasin (**39**), which was isolated from the marine sponge, *Ectyoplasia ferox*, collected on Tuxpan reef, Veracruz, Mexico. Compound **39** possesses significant cytotoxic activity against DU-145, Jurkat, MM144, HeLa, and CADO-ES1 cell lines, with IC_50_ values ranging from 2.9 to 23.5 µM, being more remarkable against DU-145 cell line with an IC_50_ value of 2.91 µM (the positive control, doxorubicin, showed IC_50_ values of 10^−7^–10^−8^ M). Moreover, it was shown that **39** induced apoptosis in DU-145 cells [164].

Three new kynurenine-bearing cyclic heptapeptides, phakefustatins A–C (**40**–**42**) (Figure 9), were isolated from the marine sponge, *Phakellia fusca*, which was collected off Yongxing Island in the South China Sea at a depth of 25 m, by using a neutral-loss scanning method based on LC-MS/MS. The structures of **40**–**42** were elucidated by HRESIMS and extensive analysis of their 1D and 2D NMR spectra, and the absolute configurations of their amino acid residues were determined by the advanced Marfey’s method. Compound **40** was identified as a retinoic X receptor-α (RXRα) modulator, which is an important nuclear receptor that could control various biological processes through inhibition of the PI3K/Akt pathway [200,201]. These findings suggested that **40** might inhibit cancer cell growth and induce apoptosis through the RXRα-mediated PI3K/Akt signaling pathway, and its pharmacophores could be kynurenine and guanidine groups [165].

A new cyclic lipopeptide, aciculitin D (**43**), was isolated from the marine sponge, *Poecillastra* sp., which was collected by dredging at a depth of 245 m near the seamount Gonsone, Japan. The structure of **43** was elucidated by HRESIMS and 1D and 2D NMR spectral analysis as well as chemical degradation. The absolute configurations of the amino acid residues were elucidated by Marfey’s method, while the absolute configurations of the sugar (lyxose) and the fatty acid (2,3-dihydroxytetradeca-4,6-dienoicacid) were established by chemical degradation and chiral LC/MS analysis. In the case of 2,3-dihydroxytetradeca-4,6-dienoicacid (Dhtda) unit, a polysaccharide-based CSP was used, allowing the conclusion that the absolute configurations of C-2 and C-3 were 2*S*,3*R* [166]. Compound **43** showed cytotoxicity against HeLa and HCT-116 cells, with IC_50_ values of 4.5 µM and 1.4 µM, respectively. The IC_50_ values of the positive control, doxorubicin, were 0.57 and 0.51 µM against HeLa and HCT-116 cells, respectively [166].

### 3.2. Antimicrobial Activity

**Figure 11:**
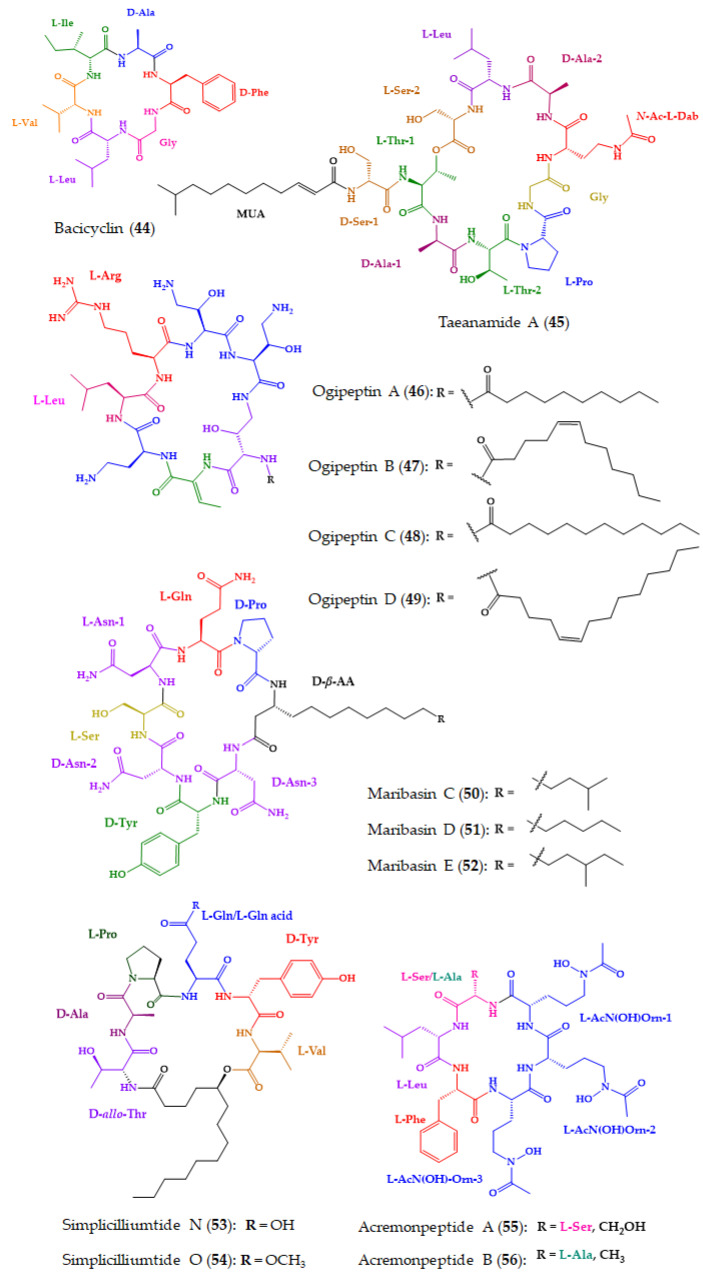

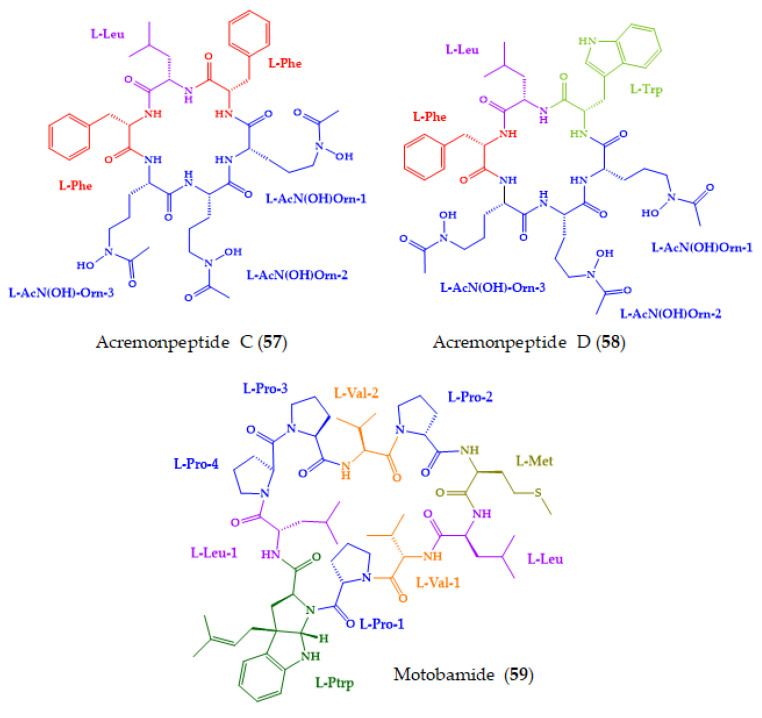
Chemical structures of marine-derived cyclopeptides with antimicrobial activity (**44**–**59**).

A number of marine-derived cyclopeptides exhibited a broad spectrum of antimicrobial activities, including bacteria, protozoa, fungi, and viruses [202,203,204]. Moreover, some of the marine cyclopeptides also showed antimicrobial activities towards several drug-resistant bacteria and fungi, making them a very promising source of novel antimicrobial agents to revert multidrug-resistance [203]. Among the 91 new marine-derived cyclopeptides isolated from 2018, 16 (**44**–**59**) were tested for their antimicrobial activity (Figure 11) and showed promising results. They were isolated from diverse marine sources including bacteria, cyanobacteria, and fungi, among others.

Bacicyclin (**44**) (Figure 11), a new cyclic hexapeptide, was isolated from a culture broth of *Bacillus* sp. strain BC028, which is associated with *Mytilus edulis*, and this was collected from the Kiel Fjord (Baltic Sea, Germany). The planar structure of **44** was elucidated by an extensive analysis of 1D and 2D NMR and HRESIMS spectra. Marfey’s method was used to determine the absolute configurations of the amino acid constituents and revealed that, in all cases, the amino acids of the building block have the l-configuration, except for alanine and phenylalanine, which had a d-configuration. Compound **44** showed antibacterial activity against the clinically relevant strains *Enterococcus faecalis* JH212 and *S. aureus* NTCT8325, with minimal inhibitory concentration (MIC) values of 8 and 12 µM, respectively. Streptomycin was used as a positive control and exhibited an MIC value of 5.24 mM against *E. faecalis* and 2.09 mM against *S. aureus*, respectively [167].

A nonribosomal lipodecapeptide, taeanamide A (**45**), was isolated from the culture of *Streptomyces* sp. AMD43, which was obtained from an intertidal-mudflat sample collected in Anmyeondo, Republic of Korea. The planar structure of **45** was elucidated by an analysis of HRESIMS and 1D and 2D spectra. The absolute configurations of the amino acid residues were determined by Marfey’s method, except for two serine residues whose stereochemistry was determined by a bioinformatic analysis of the biosynthetic gene cluster of the taeanamides. Compound **45** showed antitubercular activity against *Mycobacterium tuberculosis* mc^2^ 6230, with a concentration at which 50% of the strains were inhibited (MIC_50_) of 27 µM. Bedaquiline was used as the positive control with an MIC_50_ = 0.4 µM [168].

Ogipeptins A–D (**46**–**49**) (Figure 11), isolated from the culture broth of the marine bacterium, *Pseudoalteromonas* sp. SANK 71,903, which was collected from Ogi-machi, Sado-shi, Niigata Pref., Japan, exhibited antimicrobial activity against Gram-negative bacteria. Compounds **46**–**49** blocked lipopolysaccharide (LPS) binding to the cluster of differentiation 14 (CD14), with IC_50_ values of 4.8, 6.0, 4.1, and 5.6 nM, respectively [169,205]. LPS is a component of the cell membrane of Gram-negative bacteria and is known to induce a strong immune response [169]. LPS is well known as a bacterial endotoxin, and it is a key component for triggering sepsis or septic shock [205]. The absolute configurations of the amino acid building blocks of **46**–**49** were elucidated by the advanced Marfey’s method. This method also allowed the presence of (2*S*,3*S*) and (2*S*,3*R*) β-hydroxy-α,γ-diaminobutyric acid isomers in a ratio of 2:1 to be proved [101]. This finding was very important because chiral natural product molecules are generally assumed to be biosynthesized in an enantiomerically pure form [206].

Trikoramides B (**13**) and D (**15**) (Figure 7), isolated from the marine cyanobacterium, *Symploca hydnoides*, were also evaluated for their quorum-sensing inhibitory activity using the *Pseudomonas aeruginosa* PAO1 lasB-gfp and rhlA-gfp bioreporter strains. Compound **13** not only exhibited moderate quorum-sensing inhibitory activity, but also did not show a dose-dependent response. On the contrary, **15** exhibited moderate to significant dose-dependent quorum-sensing inhibitory effects against PAO1 lasB-gpf and rhlA-gfp bioreporter strains, with IC_50_ values of 19.6 µM and 7.3 µM, respectively. The authors hypothesized that the higher potency of **15** could be due to the brominated indole ring at the hydroxylated prenyl-tryptophan residue [155].

Three new cyclic lipopeptides, maribasins C–E (**50**–**52**) (Figure 11), were obtained from *Aspergillus* sp. SCSIO 41501, which was isolated from the Sea gorgonian, *Melitodes squamata* Nutting (Melithaidae), collected from the South China Sea, Sanya, Hainan Province. The absolute configurations of the amino acid residues were determined by Marfey’s method. Compounds **50**–**52** showed significant antifungal activity against five phytopathogenic fungal strains, *Alternaria solani*, *Curvularia australiensis*, *Colletotrichum gloeosporioiles*, *Fusarium oxysporum*, and *Pyricularia oryzae*, with MIC values of 3.12–50 μg/disc. Actidione (50 μg/disc) was used as a positive control. Comparison of the structures of the tested lipopeptides revealed that the difference in the β-amino fatty acid side chain may considerably influence the potency of the antifungal activity of this type of cyclic lipopeptides [170].

Simplicilliumtides N (**53**) and O (**54**) (Figure 11) were obtained from the deep-sea-derived fungal strain *Simplicillium obclavatum* EIODSF 020, which was isolated from a marine sediment sample collected in the East Indian Ocean. The planar structures of both compounds were established based on an analysis of HRESIMS and 1D and 2D NMR spectra. The absolute configurations of the amino acid constituents were determined by Marfey’s method. Compounds **53** and **54** showed significant antifungal activity against two phytopathogenic fungi, *Alternaria solani* (MIC values of 6.250 and 1. 562 μg/disc, respectively) and *Colletotricum asianum* (MIC values of 3.125 and 0.195 μg/disc, respectively). The positive control, ketoconazole, displayed MIC values of 6.250 and 12.5 μg/disc, against *A. solani* and *C. asianum*, respectively, while amphotericin B showed MIC values of 0.195 and 25 μg/disc against *A. solani* and *C. asianum*, respectively [171].

Acremonpeptides A–D (**55**–**58**) (Figure 11), new hydroxamate-containing cyclopeptides, were obtained from *Acremonium persicinum* SCSIO 115, which was isolated from a marine sediment sample collected in the South China Sea. The planar structures of **55**–**58** were established based on the interpretation of HRESIMS and 1D and 2D spectra, and the absolute configurations of the amino acid residues were established using Marfey’s method. Compounds **55**–**58** feature three 2-amino-5-(*N*-hydroxyacetamido)pentanoic acid (*N*^5^-hydroxy-*N*^5^-acetyl-L-ornithine) metal ion chelating moieties. Compounds **55**–**57** exhibited antiviral activity against *Herpes simplex* virus 1 with effective concentration in 50% of the population (EC_50_) values of 16, 8.7, 27, and 24 μM, respectively. The EC_50_ value of ganciclovir, a positive control, was 0.025 μM [172].

Motobamide (**59**), a new cyclopeptide containing a C-prenylated cyclotryptophan residue (Figure 11), was isolated from a marine cyanobacterium, *Leptolyngbya* sp., which was collected at Bise, Okinawa Island, Okinawa Prefecture, Japan. The planar structure of the compound was established based on an extensive analysis of 1D and 2D NMR and HRESIMS spectra. The absolute configurations of all normal amino acids were determined by a chiral LC analysis (using a ligand exchange-type CSP) of the hydrolysate after acid hydrolysis. For a prenyl-tryptophan residue, the combination of NOESY correlations and comparison of the calculated and experimental ECD spectra were applied to determine the absolute configurations of its stereogenic carbons. Motobamide (**59**) was shown to inhibit the growth of bloodstream forms of *Trypanosoma brucei rhodesiense* strains IL-1501 (IC_50_ of 2.3 μM), a causative agent of human African sleeping sickness [173].

### 3.3. Other Activities

**Figure 12:**
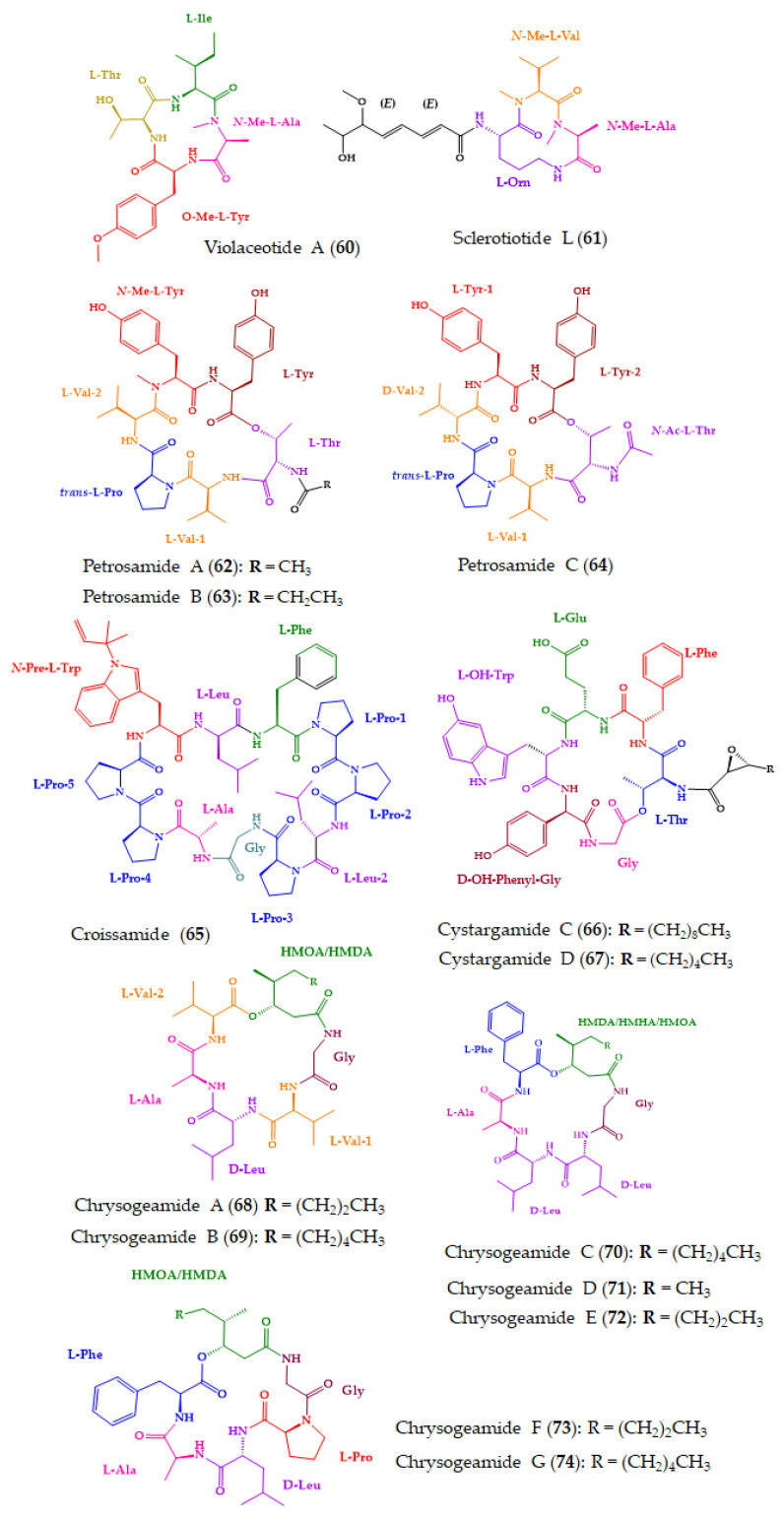
Chemical structures of marine-derived cyclopeptides with other bioactivities (**60**–**74**).

Among the 91 new marine-derived cyclopeptides isolated from various marine sources, 15 (**60**–**74**) (Figure 12) were tested for other bioactivities, some of which showed promising results.

A cyclic tetrapeptide, violaceotide A (**60**), and an aspochracin-type cyclic tripeptide, sclerotiotide L (**61**) (Figure 12), were isolated from *Aspergillus violaceofuscus*, isolated from the inner part of the marine sponge, *Reniochalina* sp., which was collected from the Xisha Islands in the South China Sea. The planar structures of both compounds were established by an extensive analysis of HRESIMS, 1D and 2D NMR spectra, and the absolute configurations of their amino acid residues were determined by Marfey’s method. Flow cytometry using the Human Inflammation Cytometric Bead Array (CBA) revealed that **60** and **61,** at a concentration of 10 µM, displayed anti-inflammatory activity against IL-10 expression of the LPS-induced THP-1 cells, with inhibitory rates of 84.3 and 78.1%, respectively [174].

Three new cyclic hexapeptides, petrosamides A–C (**62**–**64**) (Figure 12), were obtained from *Aspergillus* sp. 151304, which was isolated from the inner tissue of a marine sponge, *Petrosia* sp., collected from Yongxing Island, China. The planar structures of the compounds were elucidated by HRESIMS, and 1D and 2D spectral analysis. The configurations of the amino acid constituents were determined by the advanced Marfey’s method. Compounds **62**–**64** inhibited pancreatic lipase-mediated 4-methylumbelliferyl oleate (4-MUO) hydrolysis in a dose-dependent manner, with IC_50_ values of 7.6, 1.8, and 0.5 μM, respectively. The positive controls, isoginkgetin and orlistat, showed IC_50_ values of 2.90 μM and 0.75 nM, respectively. Further inhibition kinetics analyses showed that **64** inhibited pancreatic lipases in a non-competitive manner, while molecular dynamics simulation revealed that it could bind to pancreatic lipase at the entrance of the catalytic pocket [175].

A new proline-rich cyclopeptide containing prenylated tryptophan, croissamide (**65**) (Figure 12), was isolated from a marine cyanobacterium, *Symploca* sp., which was collected at Minna Island, Okinawa, Japan. The structures and sequence of amino acid residues were elucidated by interpretation of HRESIMS and 1D and 2D NMR spectra. The configurations of the amino acid residues were established by a chiral LC analysis, using a ligand exchange-based CSP column, of the hydrolysate resulting from the acid hydrolysis of **65**. Compound **65** showed weak inhibitory activity against NO production in LPS-stimulated RAW 264.3 cells, with an inhibition of 41.5% at 30 mM [176].

Cystargamides C (**66**) and D (**67**) (Figure 12), new lipodepsipeptides bearing six amino acids with an epoxy fatty acid side chain, were isolated from a marine actinomycete strain *Streptomyces* sp. JMS132, isolated from tidal flat sediment samples which were collected at Beolgyo, South Korea. The planar structures of the compounds were established by an extensive analysis of 1D and 2D NMR and HRESIMS spectra. The absolute structures of **66** and **67** were determined by a comparison of their circular dichroism curves with that of the previously reported cystargamide B, whose absolute configurations of its amino acid residues were determined by advanced Marfey’s methods. The absolute configurations of the stereogenic carbons of the epoxide ring of the fatty acid side chains in **66** and **67** were determined by ROESY correlations and the value of coupling constant of the vicinal protons. Compounds **66** and **67** were evaluated for their antioxidant properties through their 2,2-diphenyl-1-picrylhydrazyl (DPPH) and 2,2-azino-bis (3-ethylbenzothiazoline-6-sulfonic acid) (ABTS) radical scavenging effects. Vitamin C was used as a positive control. Compounds **66** and **67** showed significant scavenging activity on DPPH free radicals in a dose-dependent manner, with **66** able to decrease DPPH free radicals by ca. 53% at a concentration of 200 mg/mL. Compound **67**, at a concentration of 200 mg/mL, decreased ABTS free radicals by 100%, which is comparable to vitamin C [177].

By combining LC-MS/MS-dependent molecular networking and ^1^H NMR techniques, it was possible to identify seven new cyclohexadepsipeptides, chrysogeamides A–G (**68**–**74**) (Figure 12), from a coral-derived fungus, *Penicillium chrysogenum* CHNSCLM-0003. Compounds **68**–**74** exhibited angiogenesis towards zebrafish embryo at 1.0 μg/mL, with **68** and **69** promoting the cavity of blood vessels without toxicity in embryonic zebrafish at 100 μg/mL. Chrysogeamide D (**71**) comprises a rare 3-hydroxy-4-methylhexanoic acid moiety, which was first discovered to derive from marine-derived organisms. The absolute configurations of the amino acid residues were established by an HPLC-DAD and UPLC-MS analysis of the acid hydrolysate derivatized with Marfey’s reagent. Interestingly, isotope-labeling feeding experiments indicated that d-Leu could be isomerized from l-Leu [178].

### 3.4. No Demonstrated Biological Activity

**Figure 13:**
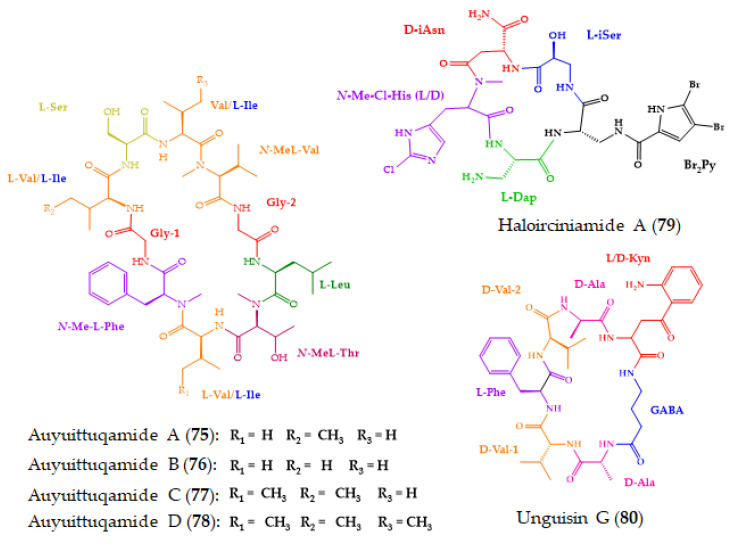

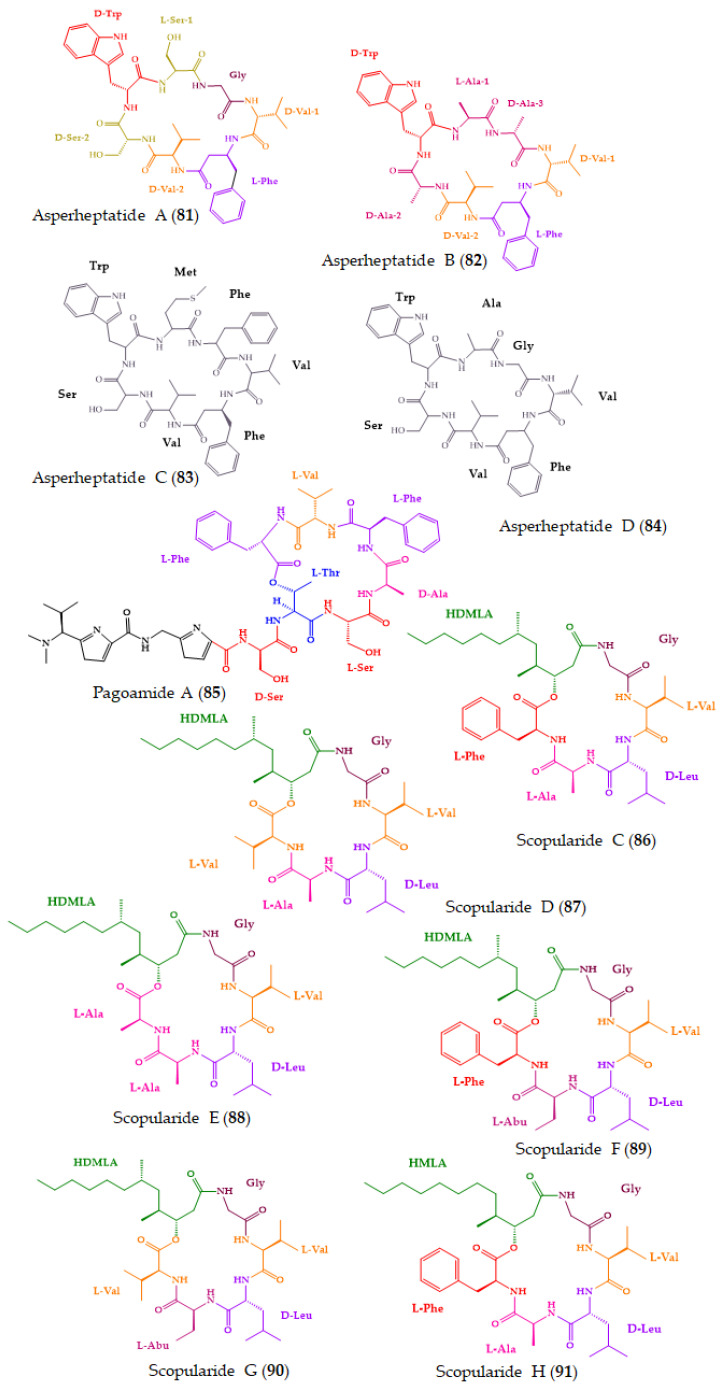
Chemical structures of marine-derived cyclopeptides that demonstrated no biological activity (**75**–**91**).

Among the 91 new marine-derived cyclopeptides reported herein, 17 (**75**–**91**) (Figure 13) were devoid of any activities in the assays performed. Consequently, these compounds should be evaluated for their potential biological effects in other available assays.

A marine-derived fungus, *Sesquicillium microsporum* RKAG 186, obtained from a marine sediment collected at low tide from the intertidal zone of Frobisher Bay, Nunavut, Canada, furnished four new cyclic decapeptides, auyuittuqamides A–D (**75**–**78**). The planar structure and the sequence of amino acids were established by HRESIMS and 1D and 2D NMR analysis. Marfey’s method was used to determine the absolute configurations of the amino acid units, except for the d-*allo*-threonine residue due to a lack of availability of a commercial standard of *N*-Me-d-*allo*-threonine. The auyuittuqamides represent a unique class of the decapeptide family due to the presence of multiple *N*-methylated amino acids. Compounds **75**–**78** showed neither cytotoxic activity against MCF-7, HTB-26, and a human epithelial keratinocyte cell line (IC_50_ values higher than 10 μM), nor antimicrobial activity at concentrations up to 128 μM [179].

Haloirciniamide A (**79**) (Figure 13), a dibromopyrrole cyclopeptide containing a chlorohistidine ring, was isolated from a marine sponge, *Ircinia* sp., which was collected in the Thousand Islands of Indonesia. The planar structure of the compound and its amino acid sequence were elucidated by an extensive 1D and 2D NMR spectral analysis as well as by (+)-HRESI-TOFMS and QTOFMS. The absolute configurations of its amino acid residues were determined by Marfey’s methods, except for *N*-methyl-chlorohistidine due to the absence of the standard amino acid. Compound **79** neither showed cytotoxicity against four human tumor cell lines, A-549, HT-29, MDA-MB-231, and PSN-1 (concentration that was able to cause 50% cell growth inhibition (GI_50_) > 1.2 × 1p^−5^ M), nor inhibited the enzyme topoisomerase I (at a concentration of 1.0 × 10^−5^ M). Moreover, **79** was unable to impair the interaction between the programmed cell death protein PD-1 and its natural ligand PDL1 [180].

**Figure 14:**
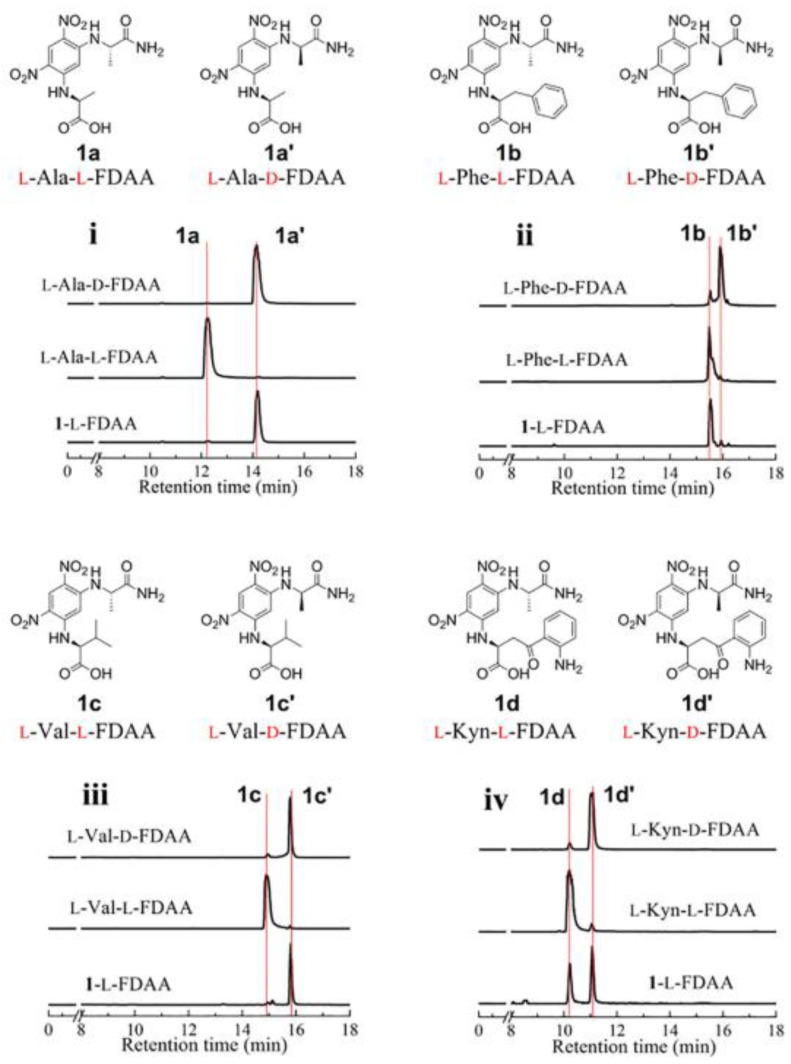
Advanced Marfey’s method using HRESIMS of unguisin G (**80**), using suitable amino acid standards: l-Kyn, l-Val, l-Ala, l-Phe. FDAA: 1-fluoro-2,4-dinitrophenyl-5-d,l-alanine amide; FDLA: 1-fluoro-2,4-dinitrophenyl-5-d,l-leucine amide (Reprint with permission from [181], Copyright (2020) ELSEVIER).

Unguisin G (**80**) (Figure 13) was obtained from the culture extract of a marine-derived fungus, *Aspergillus candidus* NF2412, which was isolated from sponge collected from the East China Sea at Ningbo, Zhejiang, Province, China. The planar structure of **80**, established by comprehensive analyses of HRESIMS and 1D and 2D NMR spectra, revealed the presence of non-proteinogenic amino acid residues, kynurenine and γ-aminobutyric acid. The advanced Marfey’s method was used to determine the absolute configurations of the amino acids of the hydrolysate obtained by acid hydrolysis of **80**, revealing not only the presence of d-alanine, d-valine, and l-phenylalanine, but also a pair of signals for l- and d-kynurenine. As shown in Figure 14, the LC-MS analysis of d/l-FDAA derivatives of acid hydrolysates of **80** with those of authentic samples showed a pair of ion signals for l- and d- kynurenine in a 1:1 ratio, indicating that this residue in **80** possesses two configurations. Even though **80** was isolated as a pair of diastereomers, attempts to separate them failed.

Compound **80** was evaluated for its antibacterial activity, by the agar diffusion method, against a series of pathogens, including *Xanthomonas oryzae* pv. *oryzicola* Swings, *Acidovorax avenae* subsp. *citrulli*, *Pseudomonas syringae* pv. *Lachrymans*, *Bacillus subtilis* CICC 10283, *Escherichia coli* ATCC 25922, *Pseudomonas aeruginosa* CICC 10351, *Micrococcus luteus* CMCC(B) 28,001, *Candida albicans* and *S. aureus* CMCC(B) 26003; however, it showed no activity against all the tested strains even at a concentration as high as 125 µg/disk [181].

By using MS/MS-based molecular networking guidance, four cyclic heptapeptides, named asperheptatides A–D (**81**–**84**), were isolated from a culture extract of *Aspergillus versicolor* strain CHNSCLM-0063, which was isolated from the gorgonian coral, *Rumphella aggregata*, collected from the Nansha Islands in the South China Sea. The planar structures and amino acid sequences of **81** and **82**, which were major compounds, were elucidated by an interpretation of 1D and 2D NMR and HRESIMS spectra. The absolute configurations of the amino acid residues of **81** and **82** were established by an LC analysis of their acid hydrolysates after derivatization with Marfey’s reagent. On the other hand, the planar structures of **83** and **84**, which were isolated as trace compounds, were characterized only by ESI-MS/MS fragmentation experiments. Compounds **81** and **82** were tested for their antitubercular activity against *M. tuberculosis* H37Ra; however, none were active (MIC values ≥ 100 µM). Rifampin and isoniazid were used as positive controls, showing MIC values of 6.25 and 12.5 nM, respectively [182].

By using MS/MS-based molecular networking guidance, pagoamide A (**85**) (Figure 13), a thiazole-containing cyclodepsipeptide comprising 11 amino acids, was isolated from laboratory cultures of a marine green alga, *Derbesia* sp., which were collected from a water depth of 12 to 18 m in Fagatele Bay, America Samoa. The planar structure of the compound and the amino acid sequence were established by 1D and 2D NMR as well as HRESIMS spectral analysis. The absolute configurations of the amino acid constituents of **85** were resolved by the advanced Marfey’s method, chiral LC, and ROESY correlations. The configuration of the *N,N*-dimethyl valine residue was determined as l by chiral LC using a ligand exchange-type CSP [(d)-penicillamine stationary phase] by comparison of the hydrolyzed product with synthetic d/l-*N*,*N*-dimethyl valine standards. Interestingly, **85** has two serine and two phenylalanine residues, and each comprises one l- and one d-residues. Density functional theory (DFT) calculations in the Gaussian program to calculate the lowest energy conformers in combination with ROESY correlations allowed the positions of d- and l-serine, and d- and l-phenylalanine residues to be assigned. The cytotoxicity of **85** against H-460 cancer cell line was evaluated; however, it was inactive at the maximum concentration tested (30 μM) [183].

By a comparative global natural product social molecular networking analysis of ×63 co-isolated fungi, new scopularides were isolated and identified: scopularides C–G (**86**–**90**) from *Beauveria* sp. CMB-F585, and scopularide H (**91**) from *Scopulariopsis* sp. CMB-F115. The chemical structures and absolute configurations of the amino acid residues were assigned by spectroscopic and C3 Marfey’s analysis, together with X-ray analyses of **86** and **91**, and biosynthetic considerations. Compounds **86**–**91** did not display significant growth inhibitory activity against a selection of Gram-positive and Gram-negative bacteria (*E. coli* ATCC 11775, *S. aureus* ATCC 9144, and three clinical isolates of methicillin-susceptible *S. aureus*, methicillin-resistant *S. aureus*, and vancomycin-resistant *E. faecalis*), a fungus (*C. albicans* ATCC 10231), and a panel of three human carcinoma cell lines (adherent SW620, NCI-H460, and HepG2) [184].

### 3.5. Final Remarks

**Figure 15:**
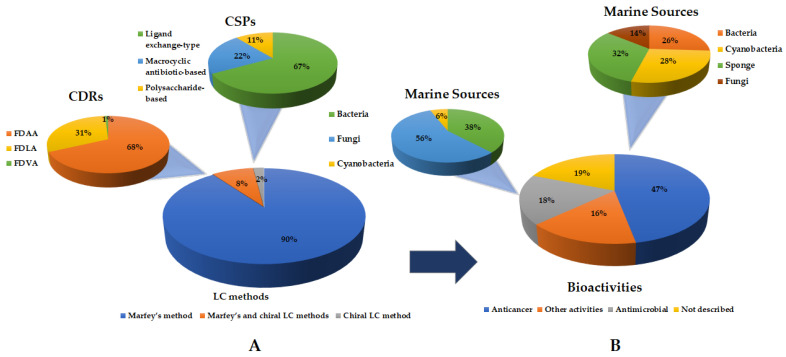
Distribution concerning: (**A**) the LC methods used for determination of the stereochemistry of marine-derived cyclopeptides, including the CSPs for chiral LC and CDRs for Marfey’s analysis; (**B**) the biological activities evaluated, highlighting the marine sources.

Regarding the stereochemical determination of cyclopeptides amino acid residues by LC methods, Marfey’s analysis was demonstrated to be the most widely used method (Figure 15), allowing the determination of the absolute configurations of 89 cyclopeptides, seven of which were associated with chiral LC. Intriguingly, there were only two marine cyclopeptides whose absolute configurations of their amino acid residues were determined only by chiral LC. As expected, the most used CDRs were FDAA, accounting for 68% of the reported analyses, while FDLA accounted for only 31% of the reported analyses. Considering the chiral LC method, the ligand exchange-type CSPs were used in most cases. There are a few reasons that may justify a massive application of Marfey’s method. First, it is a simple method which offers a better resolution when compared with a chiral LC analysis. Moreover, several derivatizing agents, such as FDAA and FDLA, are commercially available.

It is important to highlight that from January 2018 to November 2022, there were several studies on the structural revision of marine-derived cyclopeptides discovered before 2018, reporting the reinvestigation of the stereochemistry by LC and other methods, for the establishment of complete absolute configurations [138,207,208]. The focus of this review is on marine cyclopeptides, even though LC methods were chosen for the determination of the absolute configurations of the amino acid residues of the recently isolated linear peptides [209,210,211]. Surprisingly, there are still a number of reports describing the identification, structure characterization, and biological activity evaluation of novel marine cyclopeptides without stereochemistry assignments [212], or referring to only the relative configurations [213,214].

As shown in Figure 15, interesting bioactivities have been described for the marine-derived cyclopeptides, mainly anticancer (47%) and antimicrobial (18%). Nevertheless, other activities were found to account for 16% of the reported marine-derived cyclopeptides.

Regarding anticancer activity, it is important to highlight bacilohydrin A (**1**), which displayed potent cytotoxicity against three cancer cell lines with IC_50_ values of nM scale. Other relevant examples are bathiapeptides (**3**–**8**), trikoramide A (**12**), laxaphycin B4 (**22**), and microsclerodermins N (**30**) and O (**31**), with IC_50_ values ranging from 0.8 to 4.8 µM. Moreover, in addition to cytotoxicity assays, different mechanisms of action were also described. For example, epoxinnamide (**2**) exhibited antiangiogenesis activity, dentigerumycin E (**9**) showed antiproliferative and antimetastatic potential, nobilamide I (**10**) decreased cancer cell motility by inhibiting EMT effectors, ectyoplasin (**39**) was responsible for apoptotic cell death, phakefustatin A (**40**) was identified as a RXRα modulator, and asperflomide (**28**) and asperflosamide (**29**) showed inhibition of tankyrase1/2. Concerning antimicrobial activity, bacicyclin (**44**) and ogipeptins A–D (**46**–**49**) showed the most promising results of antibacterial activity, while trikoramide B (**15**) displayed quorum-sensing inhibitory effects against PAO1 lasB-gpf and rhlA-gfp bioreporter strains. Moreover, the antiparasitic activity of motobamide (**59**), antiviral activity of acremonpeptide B (**56**), and antifungal activity of simplicilliumtides N (**53**) and O (**54**) also reflected the biological versatility of marine-derived cyclopeptides.

Fungi in association with macroorganisms and other marine sources (56%) and bacteria (38%) were the main marine sources to uncover marine-derived cyclopeptides with antimicrobial activities. For those exhibiting anticancer activity, sponges (32%), bacteria (26%), and cyanobacteria (28%) were the principal marine-derived cyclopeptides suppliers (Figure 15).

Great structural diversity was found, including cyclopeptides, cyclodepsipeptides, and cyclolipopeptides, with both l- and d-configured amino acid residues. Nevertheless, for some peptides, such as tutuilamides A–C (**19**–**21**), petrosamide A (**62**), and croissamide (**65**), all the amino acids were in the l-configuration. In addition, some interesting stereochemical features were observed. For example, pagoamide A (**85**) and unguisin G (**80**) contain amino acids that appear twice in the molecule, but with opposite configuration.

Some of the peptides contained remarkable structural elements, such as non-proteinogenic amino acids. For example, a cinnamoyl moiety in epoxinnamide (**2**), polythiazole moiety in bathiapeptides (**3**–**8**), kynurenine and γ-aminobutyric acid units in unguisin G (**80**), C-prenylated tryptophan in trikoramide A (**12**) and motobamide (**59**), 4-phenylvaline and dolamethylleucine in triproamide (**16**), 2-amino-2-butenoic acid and 3-amino-6-hydroxy-2-piperidone in tutuilamides A–C (**19**–**21**), a *p*-ethoxyphenyl moiety at the terminus of the ω-phenyl-polyhydroxylated β-amino acid unit in microsclerodermins N (**30**) and O (**31**), Apoa residue in theonellamide J (**32**), 5-*cis*-Apoa-theopalauamide (**33**), and theonellamide K (**34**), β-OH Dabs in ogipeptins A–D (**46**–**49**), and chlorohistidine in haloirciniamide A (**79**), among others.

## 4. Conclusions

Ninety-one cyclopeptides have been isolated from marine sources over the last five years (January 2018–November 2022), and Marfey’s analysis demonstrated to be the most widely used method for the stereochemical determination of their amino acid residues. This review also describes several promising marine-derived cyclopeptides, some of which possess unique structural features, and with relevant biological activities, resulting from diverse marine sources. Despite being a promising area, the application of marine bioactive cyclopeptides is still lacking. Therefore, further research in bioactive cyclopeptides application is necessary to better understand their potential and applicability.

## Figures and Tables

**Table 1:** Structure, LC method for absolute configurations assignment to amino acid residues, and biological activities of new cyclopeptides (**1**–**91**), isolated from marine sources.

Marine Peptide	Type of Cyclopeptide	Marine Source	Chromatographic Method	Biological Activities	Ref.
Bacilohydrin A (**1**)	Cyclic lipoheptapeptide	*Bacillus* sp. SY27F from the Indian ocean Hydrothermal Vent	**Modified Marfey’s method combined with UPLC-ESIMS:** CDR: l-FDAA; Column: C18 (1.7 µm, 2.1 × 100 mm); MP: 0.05% NH_4_OH: CH_3_CN:0.01% HCO_2_H in elution gradient; Flow rate: 0.3 mL/min; Detection: ESIMS	Significant cytotoxicity against DU-145, MCF-7, and HepG2 cancer cell lines.	[148]
Epoxinnamide (**2**)	Cyclic decapeptide	Intertidal Mudflat-Derived *Streptomyces* sp. OID44	**Advanced Marfey’s method combined with LC-MS:** CDR: l-FDAA and D-FDAA; Column: C18 (5 µm, 4.6 × 100 mm): MP: CH_3_CN:H_2_O: 0.1% HCO_2_H in elution gradient; Flow rate: 0.7 mL/min; Detection: UV at 340 nm and MS	Induced QR activity in murine Hepa-1c1c7 cells. Antiangiogenesis activity in HUVECs.	[149]
Bathiapeptides A1 (**3**), A2 (**4**) and B-E (**5**–**8**)	Cyclic hexapeptides	Marine biofilm-derived *Bacillus* sp. B19-2	**Advanced Marfey’s method combined with UPLC-MS:** CDR: l-FDLA or d-FDLA; Column: C18; MP: CH_3_CN:0.05% TFA in elution gradient; Detection: MS	Cytotoxicity against Hep G2, HeLa, MCF-7, and MGC-803 cell lines.	[150]
Dentigerumycin E (**9**)	Cyclic hexapeptide	Coculture of marine *Streptomyces* and *Bacillus* strains isolated together from an intertidal mudflat	**Advanced Marfey’s method combined with LC-MS:** CDR: l-FDAA and d-FDAA; Column: Phenomenex Luna C18(2), (5 µm 4.6 × 100 mm); MP: CH_3_CN:H_2_O:0.1% HCO_2_H in elution gradient; Flow rate: 0.7 mL/min; Detection: UV at 340 nm and MS	Moderate cytotoxicity against A549, HCT-116, MDA-MB-231, and SK-HEP-1 cancer cell lines. Inhibition of MDA-MB-231 cell migration and cell invasion inhibition.	[151]
Nobilamide I (**10**)	Cyclic depsiheptapeptide	Marine-derived bacterium *Saccharomonospora* sp., strain CNQ-490	**C3 Marfey’s analysis combined with LC-ESI-MS:** CDR: l-FDAA; Column: Agilent Zorbax SB-C3 (5 μm, 4.6 × 150 mm); MP: H_2_O:MeOH:0.02% HCO_2_H in elution gradient; Flow rate: 1 mL/min; Detection: UV at 340 nm and ESI-MS	Inhibition of A549, AGS, and Caco2 cancer cell lines motility and tumorigenicity via suppressing EMT effectors and MMP2/9 expression.	[152]
Nocardiotide A (**11**)	Cyclic hexapeptide	*Nocardiopsis* sp. UR67 strain associated with the marine sponge *Callyspongia* sp.	**Marfey’s method combined with LC:** CDR: l-FDAA; Column: Gemini-NX RP-C18; MP: H_2_O:CH_3_CN in elution gradient; Flow rate: 1 mL/min; Detection: UV at 340 nm	Cytotoxicity towards MM.1S, HeLa, and CT26 cell lines.	[153]
Trikoramide A (**12**)	Cyclic decapeptide	Marine cyanobacterium *Symploca hydnoides*	**Marfey’s method combined with LC-MS:** CDR: l-FDVA; Column: Phenomenex Kinetex C18 (2.6 μm, 4.6 × 100 mm); MP: H_2_O:CH_3_CN:0.1% HCO_2_H in gradient elution; Flow rate: 0.2 mL/min; Detection: MS	Cytotoxicity against MOLT-4 and AML2 cancer cell lines.	[154]
Trikoramides B–D (**13**–**15**)	Cyclic decapeptides	Marine cyanobacterium *Symploca hydnoides*	**Marfey’s method combined with LC:** CDR: l-FDAA; Column: Phenomenex Kinetex C18 (2.6 µm, 4.6 × 250 mm,); MP: CH_3_CN: 0.05 M TFA (40:60); Flow rate: 0.5 mL/min; Detection: UV at 340 nm	Cytotoxicity against MOLT-4 cell line. QSI activity for **15** against PAO1 lasB-gpf and rhlA-gfp bioreporter strains.	[155]
Triproamide (**16**) and pemukainalides A (**17**) and B (**18**)	Cyclic depsiocta and hexapeptides	Marine cyanobacterium *Symploca hydnoides*	**Marfey’s method combined with HR-LCMS:** CDR: l-FDAA; Column; Phenomenex Kinetex C18 (2.6 μm, 4.6 × 250 mm); MP: CH_3_CN:0.05 M TFA (40:60); Flow rate: 1.0 mL/min flow rate; Detection: MS **Chiral LC:** Column: Phenomenex Chirex 3126 (d)-penicillamine (4.6 × 50 mm); CSP: ligand exchange-based; MP: 1 mM CuSO_4_:IPA (85:15); Flow rate: 1.0 mL/min; Detection: UV at 254 nm	**17** exhibited cytotoxicity against the MOLT-4 cell line.	[156]
Tutuilamides A–C (**19**–**21**)	Cyclic hexadepsipeptides	Marine cyanobacteria *Schizothrix* sp. (**19**–**20**) and *Coleofasciculus* sp. (**21**)	**Marfey’s method combined with LC-MS:** CDR: l-FDAA or d-FDAA; Column: Phenomenex Kinetex C18 (5 μm, 4.6 × 100 mm); MP: H_2_O: CH_3_CN:0.1% HCO_2_H in elution gradient; Flow rate: 0.6 mL/min; Detection: UV at 220, 254 and 280 nm, and HRESIMS	Cytotoxicity in H-460 cell line. Potent elastase inhibitory activity.	[157]
Laxaphycins B4 (**22**) and A2 (**23**)	Cyclic lipoundeca-peptides	Marine cyanobacterium *Hormothamnion enteromorphoides*	**Chiral LC-MS:** Column: Chirobiotic TAG (4.6 × 250 mm); CSP: Macrocyclic antibiotic-based; MP: MeOH:10 mM NH_4_OAc (40:60, pH 5.12) or (90:10, pH 6.0); Flow rate: 0.5 mL/min; Detection: ESIMS **Advanced Marfey’s method combined with LC-MS:** CDR: l-FDLA or dl-FDLA; Column: Phenomenex Kinetex C18 (2.6 μm, 2.1 × 100 mm) or Alltech Alltima C18 (5 μm, 4.6 × 250 mm), MP: H_2_O: CH_3_CN:0.1% HCO_2_H or MeOH:0.1% HCO_2_H, in elution gradient; Flow rate: 0.2 or 1.0 mL/min; Detection: MS	Antiproliferative effects of **22** against HCT-116 cell line, while **23** exhibited weak activity.	[158]
Sclerotides C–E (**24**–**26**) and scopularide I (**27**)	Cyclic hexapeptides (**24**–**26**) and lipodepsipeptide (**27**)	Soft coral-derived fungus *Aspergillus sclerotiorum* SCSIO 41031	**Marfey’s method combined with LC-ESIMS/MS:** CDR: l-FDAA; Column: YMC-Pack ODS-A (5 µm 4.6 × 250 mm); Mobile phase: CH_3_CN:H_2_O:0.03% TFA in elution gradient; Flow rate: 1 mL/min; Detection: UV at 340 nm and MS	**27** showed cytotoxicity against HONE-EBV cancer cell line and AChE inhibitory activity.	[159]
Asperflomide (**28**) and asperflosamide (**29**)	Cyclic pentapeptide (**28**) and depsihexapeptide (**29**)	Marine sponge-derived fungus *Aspergillus flocculosus* 16D-1	**Marfey’s analysis combined with UPLC-HRMS:** CDR: d-/l-FDLA; Column: Acquity UPLC HSS T3 (1.8 µm; 2.1 × 100 mm); MP: CH_3_CN:H_2_O in elution gradient; Flow rate: 0.4 mL/min; Detection: HRMS	Weak tankyrase1/2 inhibitory activity.	[160]
Microsclerodermins N (**30**) and O (**31**)	Cyclic hexapeptides	Deep-sea marine sponge *Pachastrella* sp.	**Marfey’s analysis combined with LC-MS:** CDR: d-FDAA or l-FDAA; Column: COSMOSIL 2.5 π NAP (2.1 mm); MP: CH_3_CN:0.45% CH_3_CO_2_H in elution gradient; Flow rate of 0.5 mL/min; Detection: MS	Cytotoxic against HeLa cells.	[161]
Theonellamides J (**32**) and K (**33**), and 5-*cis*-Apoa-theopalauamide (**34**)	Bicyclic glycoundecapeptides	Red sea sponge *Theonella swinhoei*	**Advanced Marfey’s method combined with UPLC-MS:** CDR: l,d-FDAA; Column: C18 (1.7 µm, 2.1 × 100 mm); MP: (A) H_2_O:CH_3_CN: 0.1% HCO_2_H in elution gradient; Flow rate: 0.5 mL/min; Detection: UV at 340 nm and ESIMS **Marfey’s method combined with HPLC:** CDR: FDAA; Column: LiChroCART RP-18 (5 µm, 4.6 × 250 mm); MP: aq. TFA buffer (pH 3):CH_3_CN in elution gradient; Flow rate: 1 mL/min; Detection: UV at 340 nm	Significant cytotoxicity against the HTC-116 cell line.	[162]
Fuscasins A–D (**35**–**38**)	Cyclic heptapeptides	Marine sponge *Phakellia fusca*	**Advanced Marfey’s method combined with UPLC-HRMS:** CDR: l-FDLA; Column: Acquity UPLC HSS T3 (2.1 × 100 mm, 1.8 μm); MP: CH_3_CN:H_2_O: 0.1% HCO_2_H in gradient elution; Flow rate: 0.4 mL/min; Detection: HRMS	**35** displayed growth-inhibitory activity against HepG2 cells.	[163]
Ectyoplasin (**39**)	Cyclic heptapeptide	Marine sponge *Ectyoplasia ferox*	**Marfey’s method combined with LC:** CDR: l-FDLA; Column: BridgeVR C-18 (5 µm, 4.6 × 100 mm); MP: CH_3_CN:H_2_O:0.05% HCO_2_H in elution gradient; Flow rate: 0.8 mL/min; Detection: 340 nm	Cytotoxicity against DU-145, Jurkat, MM144, HeLa and CADO-ES1 cancer cell lines. Apoptotic cell death of DU-145 cell line.	[164]
Phakefutatins A–C (**40**–**42**)	Cyclic heptapeptides	Marine sponge *Phakellia fusca*	**Advanced Marfey’s method combined with UPLC-HRMS:** CDR: l-FDLA and d-FDLA; Column: Acquity UPLC HSS T3 (2.1 × 100 mm, 1.8 μm); Flow rate: 0.4 mL/min; Detection: MS	**40** is a RXRα modulator to inhibit cancer cell growth.	[165]
Aciculitin D (**43**)	Cyclic lipopeptide	Marine sponge *Poecillastra* sp. collected in the deep-sea	**Marfey’s method combined with LC-MS:** CDR: d- or l-FDAA; Column: C18; MP: CH_3_CN:0.45% CH_3_CO_2_H in gradient elution; Detection: MS **Chiral LC:** Column: CHIRALCEL OJ-RH; CSP: polysaccharide-based; MP: CH_3_CN:0.45% CH_3_CO_2_H in gradient elution	Cytotoxicity against HeLa and HCT-116 cells.	[166]
Bacicyclin (**44**)	Cyclic hexapeptide	Marine *Bacillus* sp. strain associated with *Mytilus edulis*	**Marfey’s analysis combined with LC:** CDR: l-FDAA; Column: Gemini-NX RP-C18; MP: H_2_O:CH_3_CN in elution gradient; Flow rate: 1 mL/min; Detection: UV at 340 nm	Antibacterial activity against the *E. faecalis* JH212 and *S. aureus* NCTC 8325.	[167]
Taeanamide A (**45**)	Cyclic lipo-decapeptide	Intertidal-mudflat-derived *Streptomyces* sp. AMD43	**Advanced Marfey’s method combined with LC-MS:** CDR: FDAA; Column: Phenomenex Luna C18 (2) (5 µm, 4.6 × 100 mm); MP: H_2_O:CH_3_CN:0.1% HCO_2_H in gradient elution; Flow rate: 0.7 mL/min; Detection: UV at 340 nm and MS	Anti-tuberculosis activity.	[168]
Ogipeptins A–D (**46**–**49**)	Cyclic heptapeptides	Marine bacterium *Pseudoalteromonas* sp. SANK 71,903 by Daiichi Sankyo	**Advanced Marfey’s method combined with LC-MS:** CDR: FDLA; Column: C30 (5 µm, 4.6 × 50 mm); MP: H_2_O:CH_3_CN:0.1% HCO_2_H in gradient elution; Flow rate: 2.5 mL/min; Detection: UV at 254 nm and MS	Antimicrobial activity against *E. coli*. **46**–**49** blocked LPS binding to CD14.	[101,169]
Maribasins C–E (**50**–**52**)	Cyclic lipopeptides	Marine gorgonian-associated fungus *Aspergillus* sp. SCSIO41501	**Marfey’s method combined with LC:** CDR: FDAA; Column: YMC-Pack ODS-A (5 μm, 250 × 4.6 mm); MP: CH_3_CN:H_2_O: 0.03% TFA in gradient elution; Flow rate: 1 mL/min; Detection: UV at 340 nm	Antifungal activity against phytopathogenic fungi *A. solani*, *P. oryzae*, *C. australiensis*, *C. gloeosporioiles*, *F. oxysporum*.	[170]
Simplicilliumtides N (**53**) and O (**54**)	Cyclic hexapeptides	Deep-sea-derived fungal strain *Simplicillium obclavatum* EIODSF 020	**Marfey’s method combined with LC:** CDR: FDAA; Column: YMC-Pack ODS-A (250 × 4.6 mm, S-5 mm, 12 nm); MP: CH_3_CN/H_2_O/TFA in gradient elution; Flow rate: 1 mL/min; Detection: UV at 340 nm	Antifungal activity against phytopathogenic fungi *A. solani* and *C. asianum*.	[171]
Acremonpeptides A–D (**55**–**58**)	Cyclic hexapeptides	Marine fungus *Acremonium persicinum* SCSIO 115	**Marfey’s method combined with LC:** CDR: l-FDAA; Column: Prodigy ODS (2) (5 μm, 4.6 × 150 mm); MP: H_2_O:CH_3_CN:0.1% TFA in gradient elution; Flow rate: 1 mL/min; Detection: UV at 340	Antiviral activity against *H. simplex* virus 1, for **55** and **56**.	[172]
Motobamide (**59**)	Cyclic decapeptide	Marine cyanobacterium *Leptolyngbya* sp.	**Chiral LC:** Column: CHIRALPAK MA (+) (4.6 × 50 mm); CSP: ligand exchange-based; MP: 2 mM CuSO4, and CH_3_CN:2 mM CuSO4 (15:85); Flow rate: 1.0 mL/min; Detection: UV at 254 nm	Inhibition of the growth of bloodstream forms of *T. brucei*.	[173]
Violaceotide A (**60**), and sclerotiotide L (**61**)	Cyclic tetrapeptide (**60**) and lipotripeptide (**61**)	Marine sponge-derived fungus *Aspergillus violaceofuscus*	**Marfey’s method combined with LC-MS:** CDR: l-FDLA; Column: Waters XBridge C18 (5 µm, 4.6 × 250 mm); MP: H_2_O:CH_3_CN: 0.1% HCO_2_H in gradient elution; Flow rate: 1.0 mL/min; Detection: MS	Anti-inflammatory activity against IL-10 expression of the LPS-induced THP-1 cells.	[174]
Petrosamides A–C (**62**–**64**)	Cyclic hexapeptides	Sponge-*derived* *Aspergillus* sp. 151304	**Advanced Marfey’s method combined with UPLC-MS:** CDR: l-FDLA; Column: Waters HSS T3 (1.8 μm, 2.1 × 100 mm); MP: CH_3_CN:H_2_O: 0.1% HCO_2_H in gradient elution; Flow rate: 0.4 mL/min); Detection: MS	Pancreatic lipase inhibitory activity.	[175]
Croissamide (**65**)	Cyclic decapeptide	Marine cyanobacterium *Symploca* sp.	**Chiral LC:** Column: CHIRALPAK MA(+) (4.6 × 50 mm); CSP: ligand exchange-based; Mobile phase: different proportions of CH_3_CN:2 mM CuSO4; Flow rate: 1.0 mL/min; Detection: UV at 254 nm	Inhibitory activity against NO production in LPS-stimulated RAW 264.3 cells.	[176]
Cystargamides C and D (**66**–**67**)	Cyclic lipohexadepsipeptides	Marine actinomycete strain *Streptomyces* sp. JMS132	**Advanced Marfey’s method combined with LC-MS:** CDR: l-FDLA or d-FDLA; Column: Phenomenex C18 (5 µm, 4.6 × 100 mm); MP: CH_3_CN:H_2_O: 0.1% HCO_2_H in gradient elution; Flow rate: 0.4 mL/min; Detection: ESIMS	Antioxidant activity. **66** decreased DPPH free radicals. **67** decreased ABTS free radicals.	[177]
Chrysogeamides A–G (**68**–**74**)	Cyclic hexadepsipeptides	Coral-derived fungus *Penicillium chrysogenum* CHNSCLM-0003	**Marfey’s method combined with HPLC-DAD and UPLC-MS:** CDR: l-FDAA; Column: YMC C18 (5 µm, 2.1 × 250 mm) or ACQUITY UPLC BEH C18 (1.7 µm, 2.1 × 50 mm); MP: H_2_O:CH_3_CN: 0.1% HCO_2_H in gradient elution; Flow rate: 0.5 or 1.0 mL/min; Detection: DAD and MS	Pro-angiogenic activity towards Tg(kdrl:EGFP) transgenic zebrafish line.	[178]
Auyuittuqamides A–D (**75**–**78**)	Cyclic decapeptides	*Sesquicillium microsporum* RKAG 186	**Marfey’s method combined with LC-HRMS:** CDR: l-FDAA; Column: C18 (1.9 μm, 2.1 × 50 mm); MP: H_2_O:CH_3_CN:0.1% HCO_2_H in elution gradient; Flow rate: 0.4 mL/min; Detection: HRMS	Inactive against MCF-7 and HTB-26 cancer cell lines as well as against a human epithelial keratinocyte cell line. No antimicrobial activity was observed.	[179]
Haloirciniamide A (**79**)	Cyclic pentapeptide	Indonesian marine sponge of the genus *Ircinia*	**Marfey’s method combined with LC/MS:** CDR: l-FDAA; Column: Waters Symmetry (3.5 µm, 4.6 × 150 mm); MP: H_2_O:CH_3_CN: 0.04% HCO_2_H in gradient elution; Flow rate: 0.8 mL/min; Detection: LC/MS	Low cytotoxicity against A-549, HT-29, MDA-MB-231, and PSN-1 tumor cell lines.	[180]
Unguisin G (**80**)	Cyclic heptapeptide	Sponge-derived fungus *Aspergillus candidus* NF2412	**Advanced Marfey’s method combined with LC-HRESIMS:** CDR: l- and d-FDAA; Column: Agilent Poroshell 120 EC-C18 (2.7 μm, 3.0 × 50 mm); MP: CH_3_OH:H_2:_O:0.1% TFA in gradient elution; Flow rate: 0.5 mL/min; Detection: HRESIMS	No antimicrobial activity against a series of pathogens.	[181]
Asperheptatides A–D (**81**–**84**)	Cyclic heptapeptides	Coral-derived fungus *Aspergillus versicolor*	**Advanced Marfey’s method combined with LC:** CDR: l-FDAA; Column: C18; MP: CH_3_CN:H_2_O in gradient elution; Flow rate: 1 mL/min; Detection: UV at 340 nm	No antitubercular activity against *M. tuberculosis* H37Ra.	[182]
Pagoamide A (**85**)	Cyclic depsiundecapeptide	Cultured Marine Chlorophyte, *Derbesia* sp.	**Advanced Marfey’s method combined with LC-MS:** CDR: d-FDAA; Column: YMC-Triart C18 (5 μm, 10 × 250 mm); Detection: MS **Chiral LC:** Column: Phenomenex Chirex 3126 (d)-penicillamine (5 μm, 4.6 × 250 mm); CSP: ligand-exchange based; MP: 2 M CuSO_4_; Flow rate: 2 mL/min; Detection: MS	No cytotoxicity against H-460 cancer cell line.	[183]
Scopularides C–G (**86**–**90**) and H (**91**)	Cyclic lipopentadepsipeptides	Marine sponge-derived fungus *Beauveria* sp. CMB-F585, and *Scopulariopsis* sp. CMB-F115	**C3 Marfey’s method combined with LC-DAD and MS:** CDR: l and d-FDAA; Column: Agilent Zorbax SB-C3 (5 µm, 4.6 × 150 mm,); MP: H_2_O:MeOH: CH_3_CN: 0.1% HCO_2_H in gradient elution; Flow rate: 1.0 mL/min; Detection: DAD and ESIMS	No antimicrobial activity against a series of pathogens. No cytotoxicity against a panel of human carcinoma cell lines.	[184]

*A. solani: Alternaria solani*; A549: lung cancer cell line; ABTS: 2,2-azino-bis(3-ethylbenzothiazoline-6-sulfonic acid; AChE: acetylcholinesterase; AGS: gastric cancer cell line; AML2: acute myeloid leukemia cell line; *C. asianum*: *Colletotricum asianum*; *C. gloeosporioiles*: *Colletotrichum gloeosporioiles*; *C. australiensis*: *Curvularia australiensis*; CADO-ES1: Ewing’s sarcoma cell line; Caco2: colorectal cancer cell line; CD14: cluster of differentiation 14; CDR: chiral derivatizing reagent; CSP: chiral stationary phase; CT26: colon carcinoma cell line; DAD: diode-array detector; DPPH: 2,2-diphenyl-1-picrylhydrazyl; DU-145: prostate cancer cell line; *E. coli*: *Escherichia coli*; *E. faecalis*: *Enterococcus faecalis*; EMT: epithelial-mesenchymal transition; ESI: electrospray ionization; *F. oxysporum*: *Fusarium oxysporum*; FDAA: 1-fluoro-2-4-dinitrophenyl-5-d,l-alanine amide; FDLA: 1-fluoro-2-4-dinitrophenyl-5-d,l-leucine amide; FDVA: 1-fluoro-2-4-dinitrophenyl-5-d,l-valine amide; *H. simplex*: *Herpes simplex*; H-460: lung cancer cell line; HCT-116: colorectal cancer cell line; HeLa: human cervical carcinoma cell line; Hepa-1c1c7: murine hepatoma cell line; HepG2: hepatocellular carcinoma cell line; HT-29: colon cancer cell line; HTC-116: colon cancer cell line; HUVEC: human umbilical vein endothelial cells; IPA: 2-propanol; Jurkat: T-cell acute leukemia cell line; LC: liquid chromatography; LPS: lipopolysaccharide; *M. tuberculosis*: *Mycobacterium tuberculosis*; MCF-7: breast cancer cell line; MDA-MB-231: breast cancer cell line; MGC-803: gastric carcinoma cell line; MM144: human multiple myeloma cell line; MM.1S: multiple myeloma cell line; MMP: matrix metalloproteinase; MOLT-4: acute lymphoblastic leukemia cell line; MP: mobile phase; MS: mass spectrometry; *P. oryzae*: *Pyricularia oryzae*; PSN-1: pancreas cancer cell line; QR: quinone reductase; QSI: quorum-sensing inhibitory; RXRα: retinoic X receptor-α; *S. aureus: Staphylococcus aureus*; SK-HEP-1: liver cancer cell line; SNU638: stomach cancer cell line; *T. brucei*: *Trypanosoma brucei*; TEAP: triethylammonium phosphate solution; TFA: trifluoroacetic acid; THP-1: acute monocytic leukemia cell line; UPLC: ultra-performance liquid chromatography; UV: ultra violet.

## Data Availability

Not applicable.
